# Numerical study on failure process and ultimate state of steel bearing under combined load

**DOI:** 10.1016/j.heliyon.2020.e03764

**Published:** 2020-04-14

**Authors:** Hagere Alemayehu Gibe, Hiroki Tamai, Yoshimi Sonoda

**Affiliations:** a Department of Civil and Structural Engineering, Graduate School of Engineering, Kyushu University, Japan; b Department of Civil and Structural Engineering, Faculty of Engineering, Kyushu University, Japan

**Keywords:** Civil engineering, Structural engineering, Earthquake engineering, Structural analysis, Structural mechanics, Pin bearing, Pin-roller bearing, Failure process, Ultimate load capacity, Bridge axis, Perpendicular bridge axis, Dead load, Uplift load, FEM

## Abstract

The limit state and deformation performance of steel bearing under seismic load is one of the most critical points to consider the effective or rational design of bridge against strong ground motion. In the 2016 Kumamoto earthquake, various bridges are damaged by the earthquake. Among the components of the bridge, steel bearings are the most damaged part of the bridge, which affects the functionality of the entire bridge. Since the 1995 Southern Hyogo Prefecture Earthquake, several studies about the ultimate state of steel bearing during earthquake carried out. However, there are a few studies on analyzing the failure processes and ultimate state of steel bearing when various loads assumed at the time of the earthquake. Therefore, the study investigates the failure process and ultimate state of pin bearing and pin-roller bearing under combined load using static push-over analysis. First, the bridge axis and perpendicular bridge axis horizontal loading directions proposed depending on the actual earthquake directional behavior of the bridge. Then the analysis of each bearing conducted and clarified the failure process of each bearing that leads to failure based on the von mises stress yield criteria. Three-dimensional finite element method used to analyze the bearings. The analysis result found that set bolt and pin neck tensile failure were the probable failure mode of pin bearing, and failure mode of pin-roller bearing depends on vertical and horizontal loading direction. In the future, the result used to propose a new seismic resistance design and reinforcement method of bearings that satisfies the required performance.

## 1 Introduction

Earthquake is one of the most destructive natural phenomena that cause various damage on both human life and infrastructure. In the last three decades, failures of bridges recorded throughout the world, and each bridge failure has unique features, which is difficult to estimate the causes of damage. Some examples of bridge failure caused by earthquake in worldwide are 1994 Northridge earthquake occurred in northwest of downtown Los Angles and caused considerable damage to bridge structure [1], the 1995 Southern Hyogo Prefecture Earthquake induced collapse of pilz bridge [2], 1999 Kocaeli and Duzce earthquake in Turkey, and Chi-Chi earthquake in Taiwan occurred and induced fault rapture of bridge [3], 2003 Bam earthquake occurred in southeastern region of Iran and caused significant damage of bridge [4], 2008 Wenchuan earthquake happened in Chain and induced extensive damage of bridge [5], 2009 L'Aquila earthquake occurred in central Italy and caused collapse of bridge [6], 2010 Chile Earthquake happened and induced bridge collapse [7], and 2016 Kumamoto earthquake occurred in Japan and caused numerous bridge failure [8]. This paper focused on the recent earthquake of Japan that caused various damage of bridge in Kumamoto prefecture.

In the 2016 Kumamoto earthquake, various damage of bridges was reported. Some of the bridges that damaged by the earthquake are Okirihata bridge, Okirihata dam bridge, Kuwazuru bridge, Oginosaka bridge, Susukinohara bridge, Aso bridge, and Tawarayama bridge [9]. There are several causes that lead to the collapse of the bridge during the earthquake, bearing failure was one of the causes of bridge collapse at the time of the earthquake. According to the damage survey, it confirmed that various steel bearings damaged due to the earthquake. Investigation and analysis of bearing failure based on the damage situation of the bearings are necessary in order to understand deformation performance, failure process and ultimate resistance of the bearing at the time of the earthquake, and it's also used to propose new seismic resistance design and reinforcement method that satisfies the required performance. At the time of earthquake, it is essential to analyze the stress state acted on the bearing under combined loading condition, so quantitative description of failure bearing components used to clarify the situation of the collapse of each member of bearings based on the relationship of lateral load and the corresponding displacement by using finite element method (FEM).

**Figure 1:**
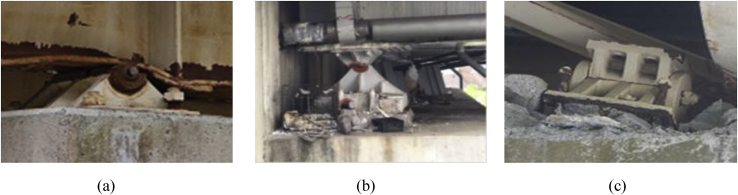
Damage situation of pin bearings due to the 2016 Kumamoto earthquake: (a) Upper seat drop out; (b) Set bolt fracture; (c) Anchor bolt fracture.

**Figure 2:**
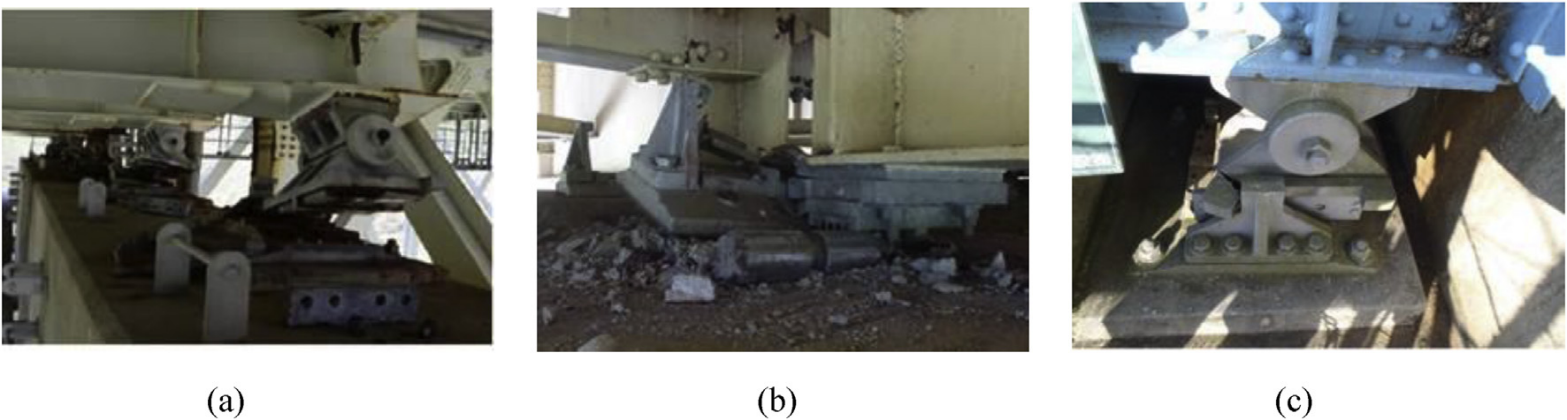
Damage situation of pin-roller bearings due to the 2016 Kumamoto earthquake: (a) Side block dropout; (b) Roller fall off; (c) Masonry plate stopper fracture.

Failure is one of the most important aspects of the material dynamic property for engineering applications. Based on the investigation of the survey various steel bearings were broken out due to seismic force that generated by the earthquake exceeded the design load of the bearing estimated by Japan Road Association design standard. The current researches are not adequate to understand the failure process of bearing under combined load at the time of the earthquake. Figure 1 shows the damage situation of pin bearing; upper seat dropped out, set bolt and anchor bolt fracture are some of the failure modes of the bearing at the time of the earthquake. Figure 2 shows damage situation of pin-roller bearing, which includes side block dropped off, roller fall off and fracture of masonry plate stopper.

There are several studies concerned on seismic response analysis, seismic design specification of the bridges, damages of bridges and ultimate state of steel bearings published by many researchers. Steelman et al. carried out experimental behavior of steel fixed bearing and implication of seismic bridge response [10], Li et al. performed ultimate shear performance and friction sliding response of laminated elastomeric bridge bearing [11], Zheng et al. emphasized seismic response prediction of multi-span steel bridge through push-over analysis [12], Gupta et al. carried out suitability of pot PTFE bearing [13], and Junichi et al. damage analysis of a bridge whose girder dropped in the 1995 Southern Hyogo Prefecture Earthquake [14], Noury et al. investigated failure analysis of martensitic stainless steel bridge roller bearings [15]. Many researchers conducted bearing ultimate and failure processes by modeling the bearing with the entire bridge but there are a few studies on the failure process and ultimate state of steel bearings conducted. Abe et al. carried out the experimental investigation of the ultimate behavior of metal bridge bearings under seismic load [16], which concerned the ultimate behavior of pin bearing, pot bearing, and roller bearing. Sumimura et al. investigated experimental study on resistance capacity of steel bearing supports in bridge under tsunami-induced loading [17], which instigated the resistance capacity of the pot bearing under tsunami-induced loading. Otsuka et al. emphasized on an experimental study on the fracture process of bearing supports by the 1995 Southern Hyogo Prefecture Earthquake [18], which investigated the fracture mechanism of pin bearing by considering vertical and horizontal loading cases in the direction of bridge axis and perpendicular bridge axis. Usami et al. investigated Experimental study on ultimate horizontal behavior of metal bearings [19], Sato et al. emphasized on effects of steel bearing performance on global seismic response of a bridge [20], which concerned on effects of pin bearing performance on bridge superstructure response during earthquake in the direction of bridge and perpendicular bridge axis. Yamahir et al. presented the analysis of steel girder damage mechanism in the 1995 Southern Hyogo Prefecture Earthquake and verification of countermeasures for seismic safety [21], which performed on nonlinear seismic response analysis of two steel bridges with continuous girders and steel piers. Tamai et al. presented a study on compactification of steel bearing by FE analysis [22], which investigated pot bearing compactification by using finite analysis method, Xiang et al. carried out experimental and numerical study on seismic sliding mechanism of laminated-rubber bearings [23], the author investigated sliding behavior of laminated rubber bearing with typical configuration, and Konstantinidis et al. Experimental investigation on the seismic response of bridge bearing [24], the author emphasized on steel-reinforced elastomeric bearing, steel-reinforced elastomeric bearing with PTEF disk, and PTEF special bearing seismic response. According to all the researchers, Steel bearings ultimate state and deformation performance and are not sufficiently understood. The finding of this research expected to clarify the failure process and ultimate limit state of pin bearing and pin-roller bearing based on analysis deformation performance and plasticization of each bearing part. The paper structured as follow; first, it reviews pieces of literature that relevant to the research. Then research material and methods (modeling and analysis technics) presented. Next discussed and summarized the analysis result. Finally, the paper concludes the estimated failure process based on analysis deformation performance and plasticization of each bearing portion.

## 2 Material and methods

### 2.1 Current design concept of steel bearings

Bearing ensure the functionality of a bridge by allowing translation and rotation to occur while supporting vertical and horizontal load. Considering the worst possible combination of movement and load is necessary for the rational design of bearing. The vertical and horizontal load transmission mechanism assumed between the members, and an appropriate space secured to generate the stress state in each member of the bearing and to ensure the response does not exceed the limit design value [25]. The current design required the following points.

The current design principle and requirement of steel bearing was based on the movement, load, restraint, serviceability, maintenance, and protection of the bearing. The current design should consider the following three points.

✓ The source of the movement (both translation and rotation).
✓ Force due to direct load, traffic load, earthquake, water, wind and temporary loads due to construction.
✓ Maximum possible protection against the environment, and allow easy access for inspection and replacement.

In Japan, the design to satisfy the performance of the bearing based on the provision of Highway Bridge Specification. Based on the load-bearing mechanism assumed according to the function required for the support parts, it is necessary to set limit status of the members constituting the bearing and the resistance characteristics value, and limit value corresponding to the limit state, and it is necessary to confirm that the limit state not exceeded for the design situation. In addition to this, the design of the bearing parts should be describe the design condition of the bearing part, items necessary for the construction and maintenance of the bridge bearing.

### 2.2 Types of bearings

The study addressed pin bearing and pin-roller bearings for analysis. All the bearings are design based on the design standard of the Japan Road Association [26]. Table 1 shows the design standard of each bearing.

**Table 1:** Design standard of the bearings.

Load	Pin (fixed) bearing	Pin-roller (movable) bearing
Total reaction force (kN)	1533	1581
Dead load reaction force (kN)	1200	1200
Bridge axial horizontal force (during earthquake) (kN)	706	289
Perpendicular Bridge axial horizontal force (during earthquake) (kN)	430	289
Lifting force (during earthquake) (kN)	243	143

#### 2.2.1 Pin bearing

**Figure 3:**
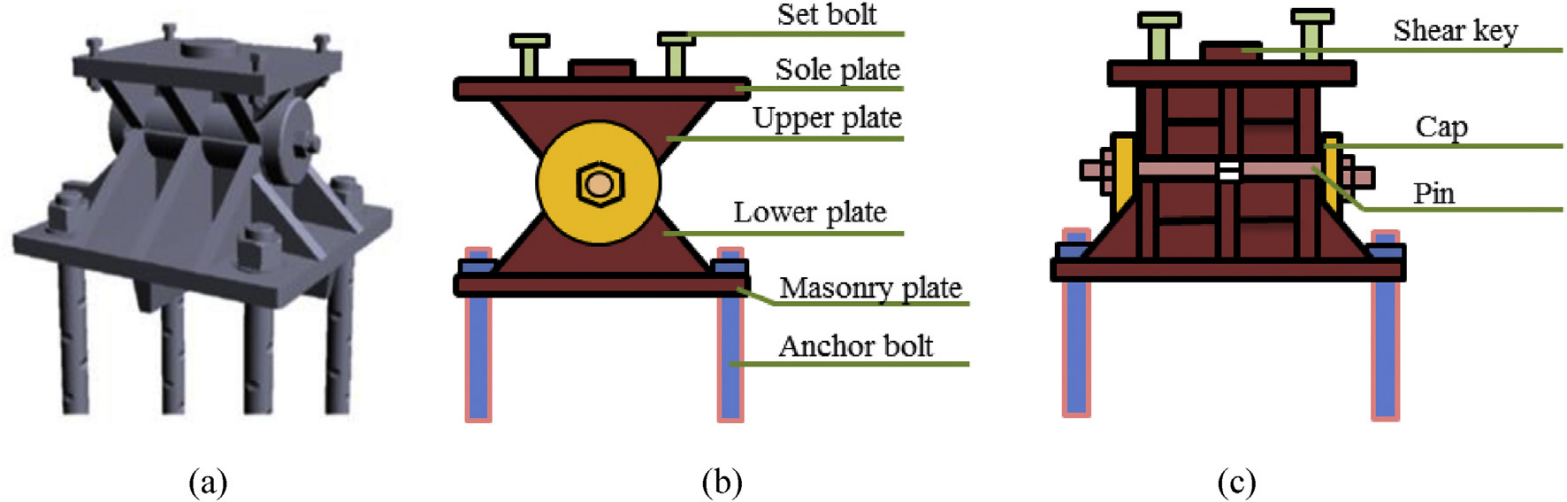
Components of pin bearing: (a) 3D model; (b) Bridge axis; (c) Perpendicular bridge axis.

Pin bearing is a type of fixed bearing that accommodates rotations using a steel pin. The pin at the top is composed of the upper and lower semi-circular recessed surface with a solid circular steel pin placed between the plates, usually, there is a cap at both ends of the pin to keep the pin from sliding off the seat and to resist uplift forces. The upper plate connected to the sole plate and the lower plate placed on the masonry plate. It used to transfer load through rotation from sole plate to masonry plate, the plates usually anchored by bolt, which resist translational movement [27] as shown in Figure 3.

#### 2.2.2 Pin-roller bearing

**Figure 4:**
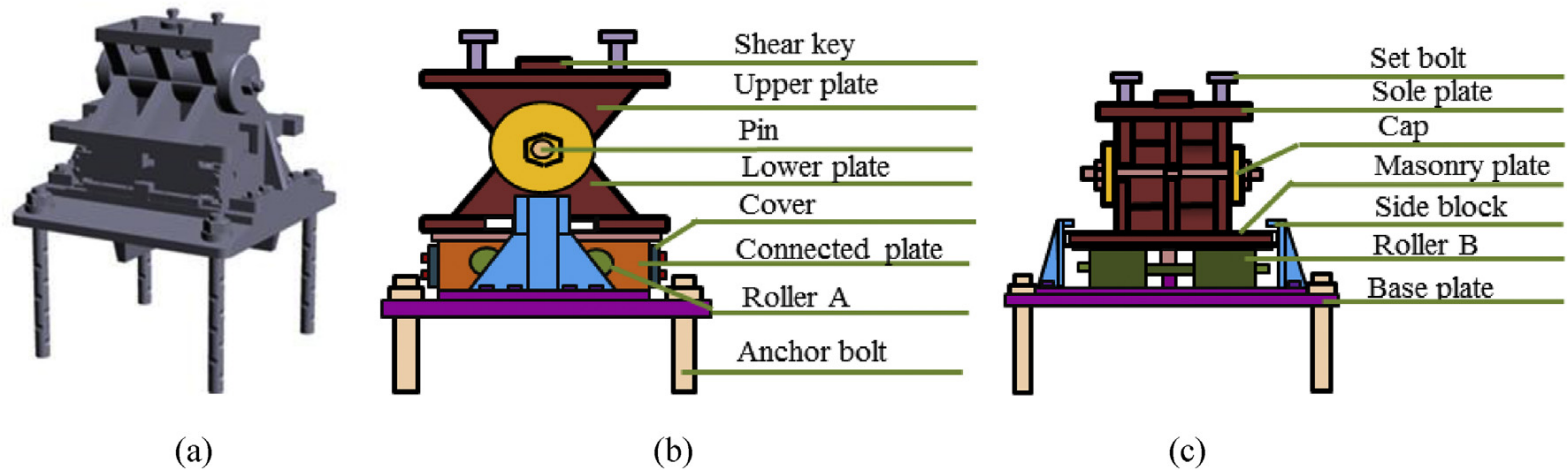
Components of pin-roller bearing: (a) 3D model; (b) Bridge axis; (c) Perpendicular bridge axis.

Pin-roller bearing is a special form of movable roller bearing in which the pin provided for easy rocking, and bottom parts of the pin placed on a series of rollers. Translational movement accommodated by the pin and rotation movement accommodated only if the rollers combined with pin as shown in Figure 4. The bearing can accommodate large movement and sliding as well as rotational movement [27].

### 2.3 Material properties of bearings

Material properties of the bearing components are the major concern in the past two decades in japan due to various bearing failure. In this study, the material properties of pin bearing and pin roller bearings selected based on Japan Road Association design Specification. Material Properties such as tensile strength, elongation (deformation), and hardening are highly dependent on the composition of the bearing. The properties listed below reflected the typical properties of each bearing part used for analysis.

All the components of the pin and pin roller bearings are made of steel as shown in Tables 2 and 3, respectively. According to the Japan Road Bridge design book, Young's modulus and poison ratio of all the parts of the bearing is the same but the yield strength is vary depending on the material types of steel.

**Table 2:** Material property of pin bearing.

Components	Material	Young's modulus, E (N/mm^2^)	Poison ratio, υ	Yield strength, $\sigma_{y}\;$(N/mm^2^)
Sole & upper plate	SCW480N	2.0 × 10^5^	0.3	275
Upper & masonry plate	SCW480N	2.0 × 10^5^	0.3	275
Set bolt	JIS B, 10.9	2.0 × 10^5^	0.3	940
Cap	SS400	2.0 × 10^5^	0.3	215
Anchor bolt	SS400	2.0 × 10^5^	0.3	215
Steel pin	S35CN	2.0 × 10^5^	0.3	305

**Table 3:** Material property of pin-roller bearing.

Components	material	Young's modulus, E (N/mm^2^)	Poison ratio, υ	Yield strength,$\sigma_{Y}$ (N/mm^2^)
Sole & upper plate	SCW480N	2.0X10^5^	0.3	275
Upper & masonry plate	SCW480N	2.0X10^5^	0.3	275
base plate	SCW480N	2.0X10^5^	0.3	275
side block	SCW480N	2.0X10^5^	0.3	275
Cap,gear, cover	SS400	2.0X10^5^	0.3	215
rock,endpice, side plate	SS400	2.0X10^5^	0.3	215
connecting plate	SS400	2.0X10^5^	0.3	215
Anchor bolt	SS400	2.0X10^5^	0.3	215
Roller A & B	C-13B	2.0X10^5^	0.3	540
Side plate 2	C-13B	2.0X10^5^	0.3	540
Steel pin	S35CN	2.0X10^5^	0.3	305
Set bolt	JIS B, 4.6	2.0X10^5^	0.3	240
Side block bolt	JIS B, 10.9	2.0X10^5^	0.3	940

### 2.4 Analysis method and modeling

#### 2.4.1 Outline of the analysis

In this study, the finite element method (FEM) is the proposed analysis method that used to clarify the failure process and ultimate limited state of the steel bearing by considering nonlinear material properties and large deformation theory under static pushover analysis. The model geometry created by an isoparametric solid element and discretized with the model domain into discrete elements. The geometrical and material properties of the bearings assigned based on the Japan Road Association design standard. Depending on the contact force and coefficient of friction at the contact surface of the bearing, the analysis assumed the penalty method and shear friction model for contact and friction problem, respectively. The contact model of large assembly that consists of multiple component is complex to understand contact condition and contact body interaction. In this study, automatic contact detection method used for modification of contact based on physical proximity of contact part. The analysis involved material nonlinearity and contact, Newton-Raphson method was used to evaluate the material nonlinearities, and residual force was used to determine the convergence. The idea of residual force depends on monitoring of nodal force contributions, and comparison of maximum force with respect to residual force. To improve analysis convergence, the sub-step automatically adjusted according to the convergence status and a cutback function was used to enhance the efficiency of the analysis results.

#### 2.4.2 Loading method

Several types of bearing fractures observed due to the 2016 Kumamoto earthquake. The major fracture of bearing classified into two, bridge axis and perpendicular bridge axis failure. According to the actual failure condition of the bearings, the analysis assumed bridge axis and perpendicular bridge axis direction horizontal loading, and dead reaction or uplift force as a vertical loading. The vertical loading which is equivalent to the structural dead reaction force or lifting force during earthquake applied on the upper surface of the sole plate except shear key, and surface of set bolt head by providing rigid surface acted like structure placed on the top of the sole plate, so the loading transferred through rigid surface. Set bolt shank and shear key of the sole plate are the two applied surface for horizontal loading. The analysis implemented a variety of horizontal load under constant vertical load.

#### 2.4.3 Analysis load cases

**Dead loading,** the vertical load of the bearing assumed based on dead load or self-weight of supper structure, which is the weight of the deck including girder and slab [28]. The analysis considered the reaction force of the bridge supper structure on the bearing.

**Seismic loading,** lateral load arise from earthquake generated either in the longitudinal or transverse direction of the bridge, in which the study considered the seismic loading bridge axis and perpendicular bridge axis respectively. The horizontal loading to the bearing resulting from the restrain comes from the analysis of the structure [28].

**Figure 5:**
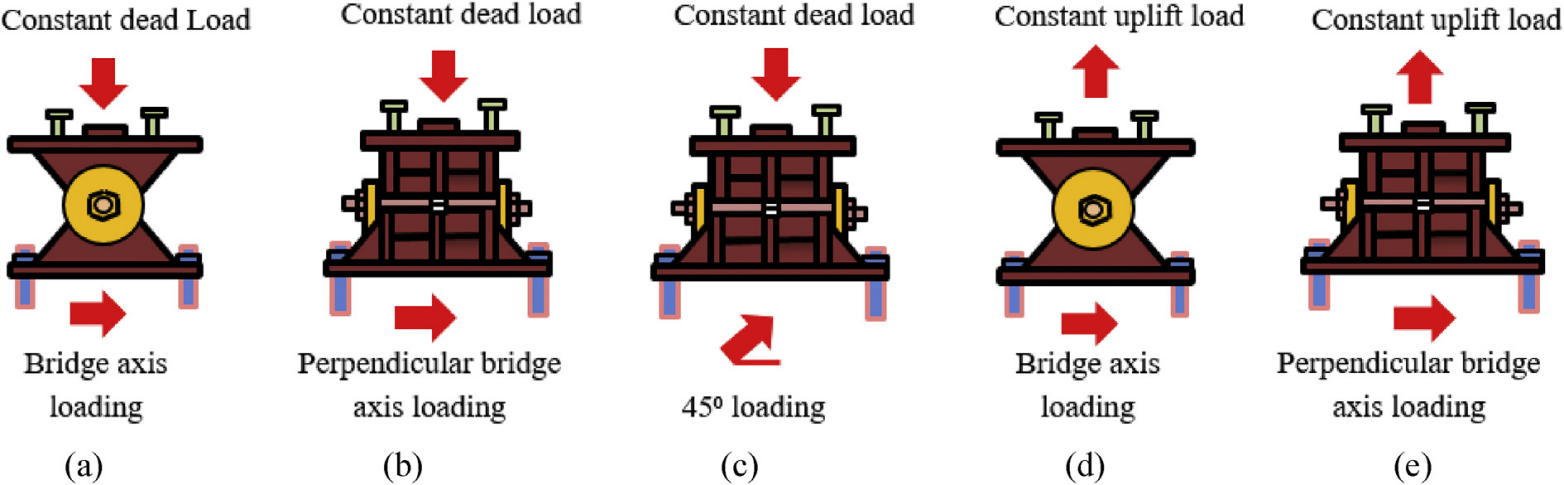
Example of pin bearing analysis load case: (a) Case 1; (b) Case 2; (c) Case 3; (d) Case 4; (e) Case 5.

**Uplift loading,** the vertical forces exerted by bridge decks and other structures on their supports are not always downward [28]; uplift can occur for a variety of reasons. The study considered the uplift load arises from vertical ground acceleration due to earthquakes. The analysis set five load cases depending on the direction of vertical and horizontal loading as shown in Table 4. Figure 5 shows load cases of pin bearing that used to anticipate failure process and ultimate state of the bearing.

**Table 4:** Analysis load cases.

Cases	Vertical loading	Horizontal loading
Bridge axis loading (Case 1)	Constant dead load	Increase force-displacement
Perpendicular bridge axis (Case 2)	Constant dead load	Increase force-displacement
45^0^ loading (Case 3)	Constant dead load	Increase force-displacement
Bridge axis loading (Case 4)	Constant uplift load	Increase force-displacement
Perpendicular bridge axis (Case 5)	Constant uplift load	Increase force-displacement

The effect of the horizontal loading examined by comparing the result of case 1, 2 and 3, and the result of case 3 and 4 used to grasp the effects of vertical load. Table 5 shows dead load and uplift load of the bearings used for analysis.

**Table 5:** Dead and lifting load of the bearings.

Type	Dead Load (kN)	Uplift load (kN)
pin bearing	1200	243
Pin-roller bearing	1200	143

### 2.5 Numerical model development

In the condition that the configuration of the body has nonlinear material properties, the method used to solve the analysis is usually the element discretization method. In this study, the numerical model developed for both longitudinal and transverse direction using multiple linear material properties and characteristics of each bearing.

#### 2.5.1 Modeling of the bearings

For simplicity, the full and half model of the bearings are used. Table 6 summarized the total number of elements and nodes of each bearing used in the analysis. All the constituent elements are hexagonal solid element in order to grasp the geometry properly.

**Table 6:** Total number of elements and nodes.

Type	Case	No.elements	No.nodes
Pin bearing	1,3,4	122,636	153,778
	2,5	61,318	76,889
Pin-roller bearing	1–5	88,118	123,096

**Figure 6:**
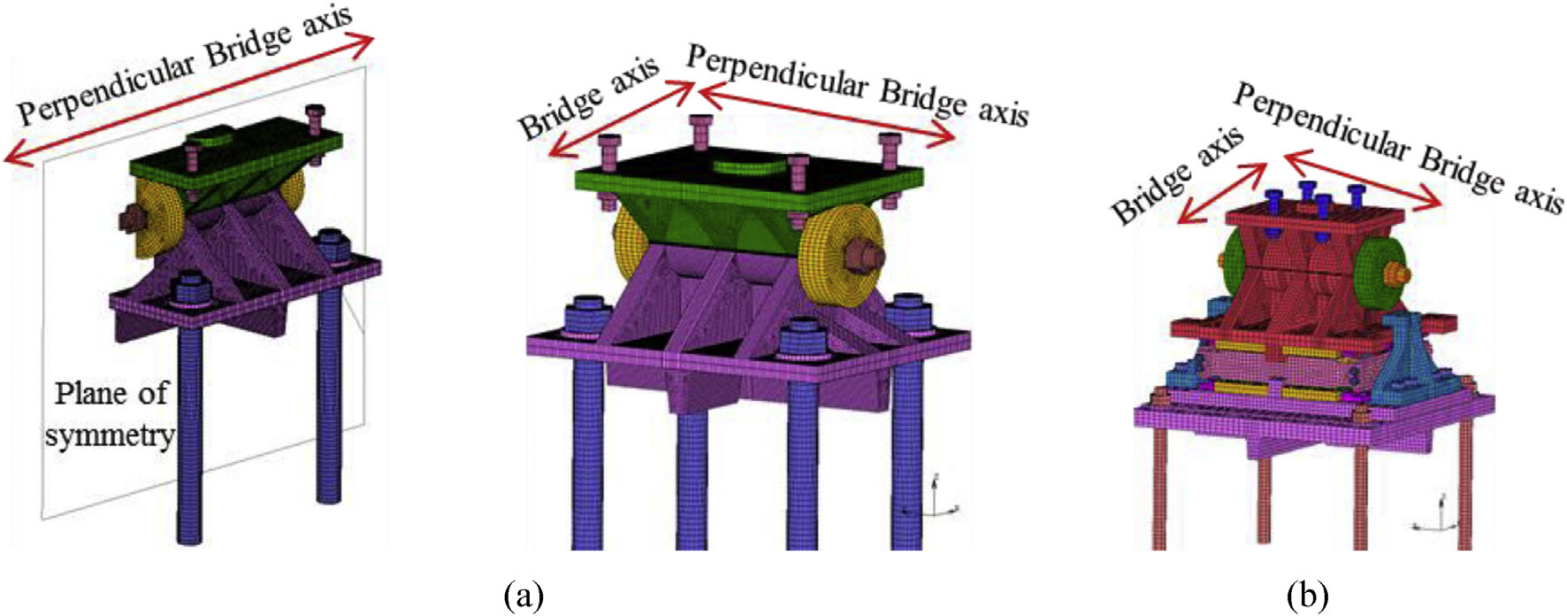
Numerical modeling of bearings: (a) Pin bearing; (b) pin-roller bearing.

Pin bearing used both half and full model for analysis. Case 1, 3 and 4 are used full model but case 2 and 5 are used a half model of the bearing by using symmetry as shown in Figure 6(a). Pin-roller used only full model for analysis, so case 1 up to 5 used full model of the bearing as shown in Figure 6(b).

#### 2.5.2 Boundary condition

In modeling, boundary condition is the most important aspect of analysis. The analysis assumed different types of boundary conditions in order to ensure accurate and expected results of bearing. Each bearing boundary condition elucidate below.

**Figure 7:**
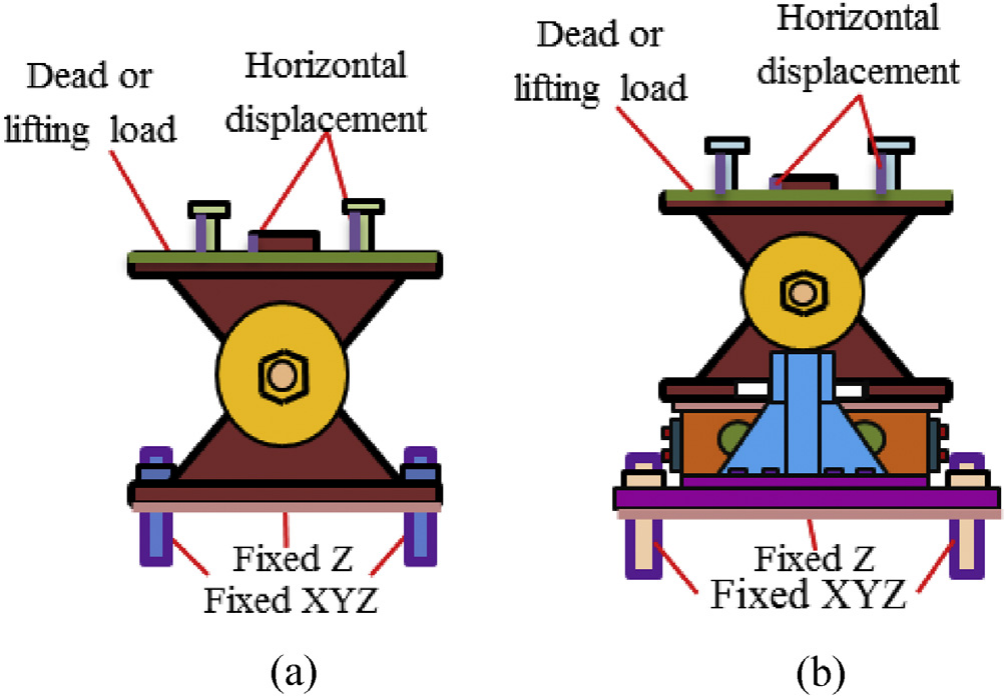
Boundary condition of bearings:(a) Pin bearing; (b) Pin-roller bearing.

Figure 7 (a) and (b) show the boundary condition of the pin bearing and pin-roller bearing, respectively. Depending on the actual installation of bearing, the anchor bolt restrained in all translation and rotation (all degree of freedom restrain) by considering actual installed bolt inside the pier or abutment. The bottom surface of the masonry plate restrained on vertical direction only by assuming the plate directly placed on concrete. The loading elucidated in section 2.4.2 (loading method) considered as another boundary condition of the analysis. The bearing considers the rigid surface to set bolt in order to prevent rotation of set bolt when the horizontal loading acts in the direction perpendicular to the bridge axis.

### 2.6 Analysis consideration

#### 2.6.1 Material constitutive law

In the nonlinear static analysis that involves material nonlinearity and large strain, it is important to consider the incremental formulation of the equation of motion for analysis. Marc software has two advanced analysis options for large strain, which is total and updated Lagrangian formulations. The reference of total lagrangian configuration used t = 0 and updated lagrangian configuration used t = n+1. Total Lagrangian formulation is not convenient for the plasticity behavior because it generates large rotation and small strain, so in this study updated Lagrangian formulation was used for analysis [29].

**Figure 8:**
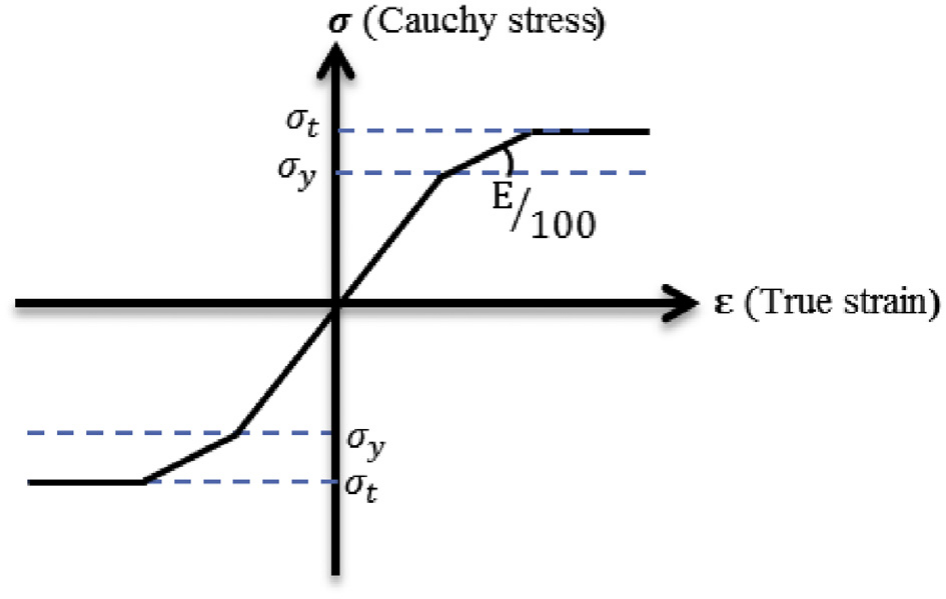
Stress-strain curve.

For isotropic material, the von Mises yield condition is preferable for analysis of steel bearing, so the plasticity of the material in the analysis controlled by von mises yield criteria. The von Mises criteria states that yield occurs when the equivalent stress (σ) equals the yield stress (σy) [29]. The material constitutive equation used for analysis based on von mises yield criteria. Figure 8 shows the stress-strain of the material. From the figure, it can be seen that assumed constitutive relationships of the material is depending on the Cauchy stress and True strain with a hardening coefficient of 1/100. E is the young's modulus of every material. The stress became constant when the von mises stress equivalent to the tensile strength of the material.

#### 2.6.2 Contact model

In this study, the contact problem (penetration and separation) of the bearings treated by penalty method. Touching and glue contact are the types of contact for deformable and rigid bodies, in which touching allows relative sliding of the bodies in the contact interface, and glue suppresses all the relative movement between the bodies [28].

**Figure 9:**
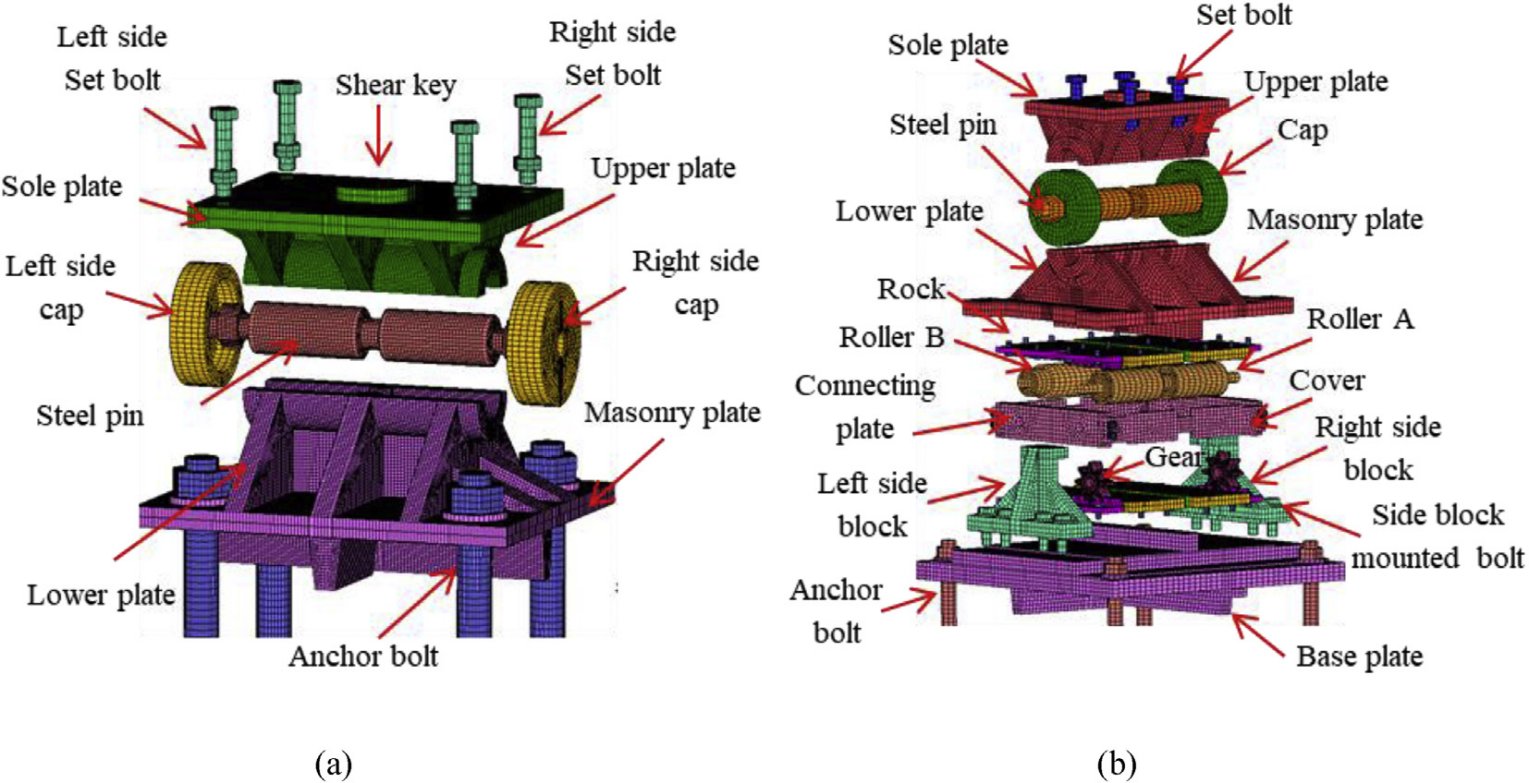
Schematic view of bearings assembly: (a)Pin bearing; (b) Pin-roller bearing.

Figure 9 (a) shows the Schematic view of pin bearing, the bearing adapted touching surface contact for all parts. Figure 9 (b) presents the Schematic view of pin-roller bearing; the surface contact adapted touching and glue contact. Glued contacted surfaces of the bearing are connecting plate glued with cover-mounted bolt, and masonry plate glued with a rack, end piece, and side plate mounted bolts.

The penalty method is an alternative method to satisfy the incompressibility constraints. The method allowed small penetration, and contact force is proportional to contact stress [27].

(1) Kc=K+χKp

where χ is a large number typically between 10^5^ to 10^9^ and Kp is the penalty matrix, Kp can be written as

(2) $$\text{Kp}=\text{C}{\text{M}}_{\text{p}}^{-1}{\text{C}}^{\text{T}}$$

where C and M_p_ are a function of geometry and shape function, respectively.

Maximum contact stress is a key design criterion for bearings when two bodies with curved surfaces are in contact under a force, point or line, and surface contact between these bodies' changes to area contact, and 3D stresses are developed.

✓ Contact stress for a cylinder in the inner cylinder.

(3) $$\text{Contact}\;\text{stress,}\;\text{σ}=\frac{Radial\;load}{projected\;area}=\frac{\text{F}}{\text{DxL}}$$

where σ is contact stress (N/mm^2^), F is radial load (N), D is the diameter of contact surface (mm), L is the length of the contact surface.

✓ Contact stress for roller in contact with a flat plate.

where E is young's modulus (N/mm^2^), F is load (N), R is radius (mm), υ is poison ratio and L is the length (mm).

(4) $$\text{Contact}\;\text{width,}\;\text{w}=\sqrt{\frac{32\text{F}\left(1-{\text{υ}}^{2}\right)\text{R}}{\text{πLE}}},\;\frac{1}{{\text{E}}^{\text{∗}}}=\frac{1-{\text{υ}}_{1}^{2}}{{\text{E}}_{1}}+\frac{1-{\text{υ}}_{2}^{2}}{{\text{E}}_{2}}$$

(5) $$\text{Contact}\;\text{stress,}\;\text{σ}=\sqrt{\frac{{\text{PE}}^{\text{∗}}}{\text{πR}}}$$

✓ Contact stress for surface contact.

(6) $$\text{Contact}\;\text{stress,}\;\text{σ}=\frac{\text{F}}{\text{A}}$$

where F is load (N) and A is the contact area.

#### 2.6.3 Friction model

Friction depends on contact force as well as the coefficient of friction at contact surfaces [29]. In this study, the shear bilinear model has been adapted for analysis based on relative tangential displacements. The bilinear model adapted based on the assumption that the shear stress in a node is proportional to the applied shear force. The shear based model states that the frictional stress is a fraction of the equivalent stress σ in the material:

(7) $$\left|{\text{σ}}_{\text{t}}\right|<\text{μ}\frac{\text{σ}}{\sqrt{3}}\;\text{and}\;{\text{σ}}_{\text{t}}=-\text{μ}\frac{\text{σ}}{\sqrt{3}}.\text{t}$$

where μ is the friction factor.

The bilinear model has been adapted based on the assumption that the shear stress in a node is proportional to the applied shear force. Similar to the friction stress limit, the shear stress due to friction is limited by:

(8) $${\text{σ}}_{\text{t}}=\min\left(\text{μ}{\text{σ}}_{\text{n}},\;\text{μ}\frac{\text{σ}}{\sqrt{3}}\right)$$

#### 2.6.4 Validation of analysis model

The analysis involved so many contact problems, so verification of the analysis result is necessary. Since it is difficult to validate the entire bearing, sample modeling was prepared to verify the analysis. The verification of pin and pin-roller bearing conducted by using a cylinder in inner cylinder contact and cylinder on flat plate contact, respectively.

For pin bearing, the theoretical value of the contact stress calculated by using Eq. (3), the load is 1.2 × 10^6^ N, the diameter is 75mm, and the contact length is 360mm.

$$\;\text{Contact}\;\text{stress,}\;\text{σ}=\frac{\text{F}}{\text{DxL}}=\frac{1.2\text{X}10^{6}}{75\text{∗}360\;}=44.45\;{\text{N/mm}}^{2}$$

**Figure 10:**
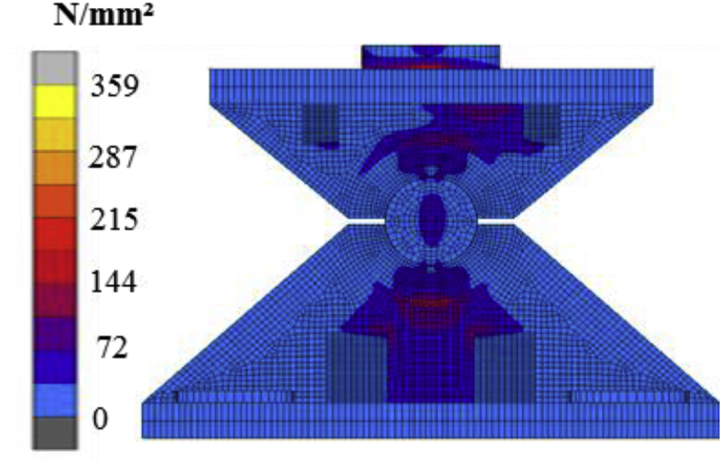
Equivalent stress distribution (Cylinder in inner cylinder contact).

The analysis contact stress evaluated by using the lower plate and cylindrical part of the steel pin as shown in Figure 10. The figure shows equivalent stress distribution under a vertical load of 1.2 × 10^6^ N. Contact stress between lower semi-circular recessed surfaces and solid circular steel pin is 46.672 N/mm^2^. The theoretical and the analysis output shows closed results; this shows the reliability and consistency of analytical results.

For pin-roller bearing, the theoretical value of the contact stress calculated by using Eq. (4) and Eq. (5) under the consideration of load 1.2 × 10^6^ N, radius 40mm, young's modulus 2 × 10^5^ N/mm^2^, poison ration 0.3, and length of the contact 285mm.

$$\frac{1}{{\text{E}}^{\text{∗}}}=\frac{1-{\text{υ}}_{1}^{2}}{{\text{E}}_{1}}+\frac{1-{\text{υ}}_{2}^{2}}{{\text{E}}_{2}}=\frac{1-0.3^{2}}{2X10^{5}}+\;\frac{1-0.3^{2}}{2X10^{5}}=\frac{1.82}{2.0X10^{5}},\;{\text{E}}^{\text{∗}}=\frac{2.0X10^{5}}{1.82}=1.1X10^{5}\text{N}/{\text{mm}}^{2}$$

$$\text{Contact}\;\text{stress,}\;\text{σ}=\sqrt{\frac{{\text{PE}}^{*}}{\text{πR}}}=\sqrt{\frac{4211*1.1*10^{5}}{\text{π}*40}}=1919.82\;\text{N}/{\text{mm}}^{2}$$

**Figure 11:**
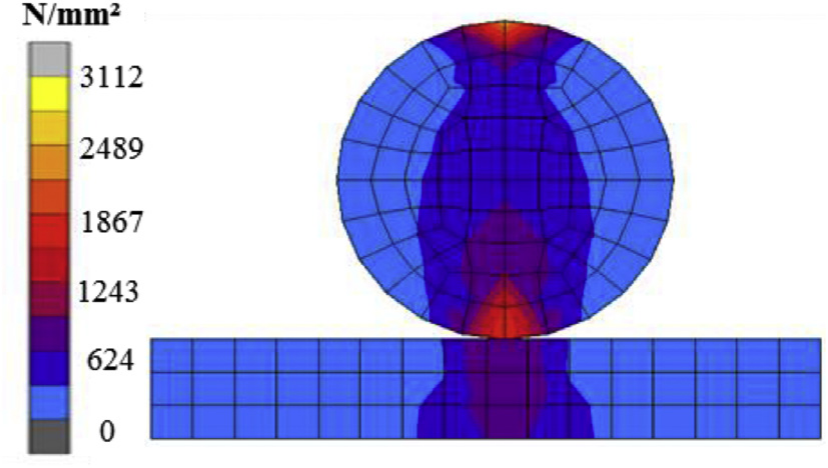
Equivalent stress distribution (cylinder on flat plate contact).

The analysis contact stress evaluated by using roller and flat plate as shown in Figure 11. The figure shows equivalent stress distribution under a vertical load of 1.2 × 10^6^N. Contact stress between roller and plate is 1867.284N/mm^2^. The theoretical and the analysis result are closed; this shows the reliability and consistency of analytical results.

## 3 Results and discussion

To predict the failure process of the entire bearing, each bearing part plasticization checked under horizontal and vertical loading. The von Mises yield criterion used to identify parts that initiated yielding under loadings. Here the checking mechanism depends on whether or not the equivalent von Mises stress exceeded the yield strength (stress) of the bearing body. The detail analysis result and discussion of each bearing describe below.

### 3.1 Pin bearing

#### 3.1.1 Bridge axis horizontal loading (Case 1)

**Figure 12:**
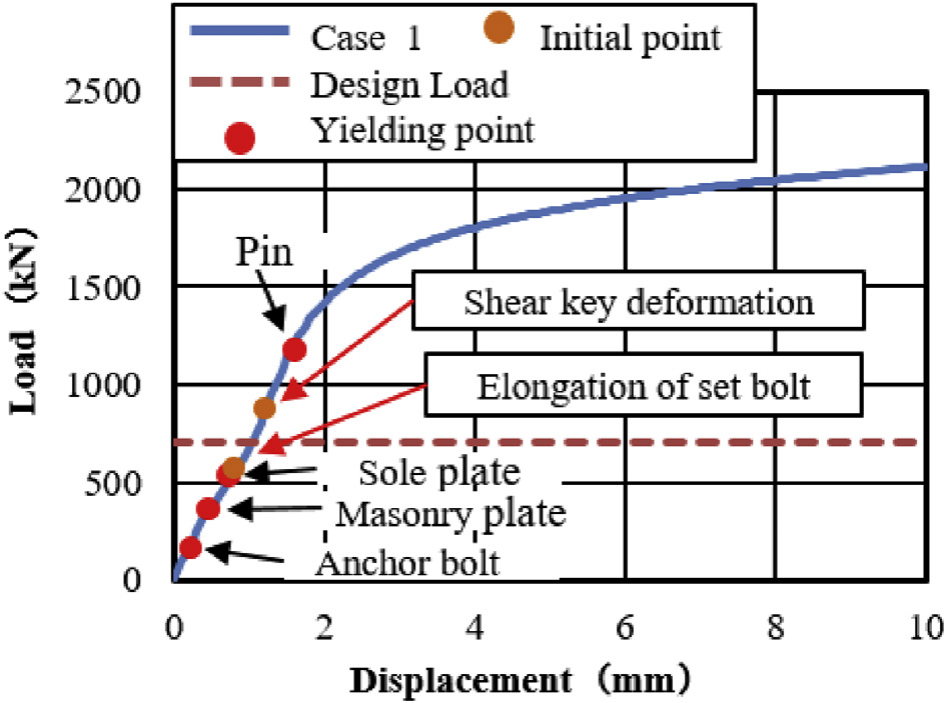
Load-Displacement curve (case 1).

**Figure 13:**
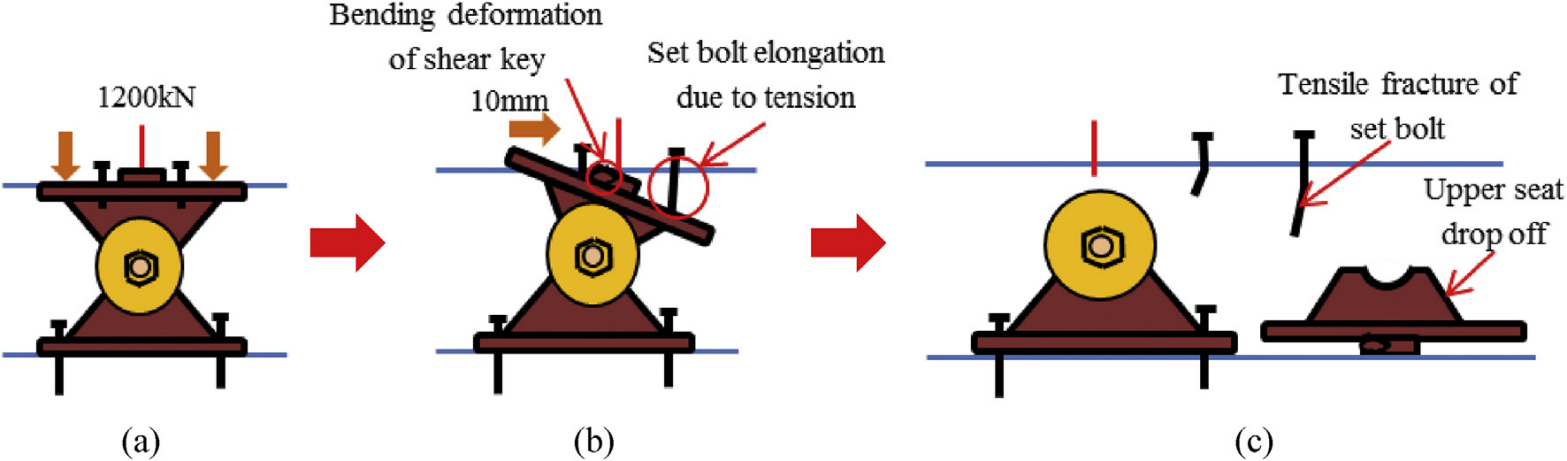
Failure process (Case 1): (a)Initial loading status; (b) constant dead load and horizontal loading; (c) Failure final status.

**Figure 14:**
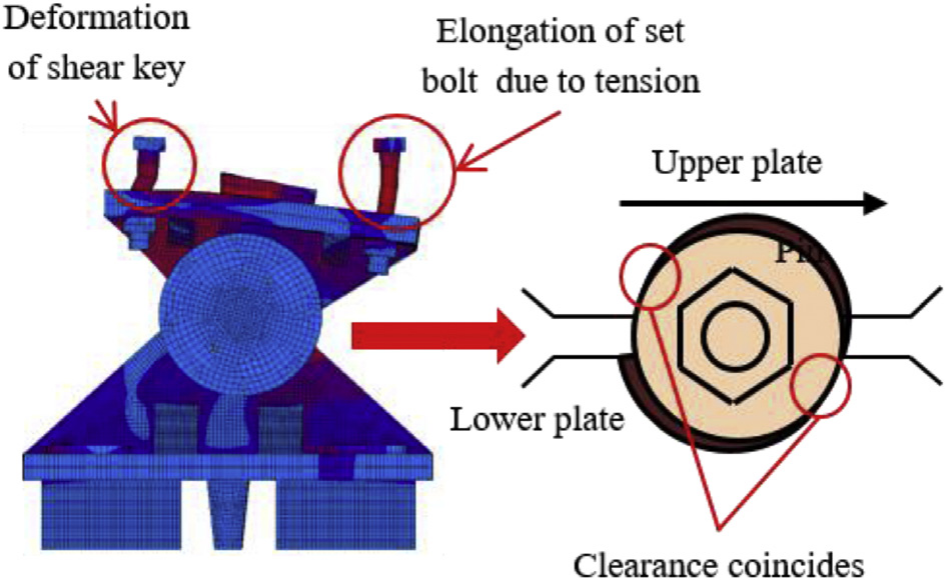
Deformation performance.

The load-displacement relationship introduced by horizontal bridge axis loading as shown in Figure 12. From the curve, the maximum load capacity of the bearing before 10mm displacement is about 2100kN, and plasticization initiated below the expected design load except the steel pin. Here, the capacity of the bearing not reached at the ultimate state due to the bearing was not under failure when the analysis completed, and the stress introduced on the set bolt not exceeded the ultimate strength of the right side set bolt (940kN). As the horizontal loading increase in the direction of the bridge axis, contact of the upper plate and lower plate semi-circular recessed surface with solid pin confirmed at 130kN and 200kN, respectively. The recessed surface of the plates introduced yielding due to the stress generated by the contact exceeded the design yield strength of the plates (275MPa). Contact between masonry plate and anchor bolt also confirmed at 200kN, and the anchor bolt subsequently initiated plasticization. When the external load exceeded the frictional load of the upper seat, the upper seat start to rotate around the pin, and caused distortion on the right and left side set bolts. As the rotation of the upper seat gradually increased, the masonry and sole plate generated yielding around 330kN and 550kN, respectively. The steel pin also initiated plasticity at 1200kN on the upper surface of the pin due to the stress introduced between the interfaces exceeded the pin yield strength (305kN). Figure 13 shows the failure process of bridge axis loading. From the figure, upper and lower weir detachment generated due to the rotation of the upper seat in the direction perpendicular to the bridge axis, and caused slipping of the upper seat in the direction of the loading. The slipping and rotation of upper seat caused plate coincident with pin, elongation of the right side set bolt (Initiated at the vicinity of 570kN), bending deformation of shear key (Induced at the vicinity of 850kN) as shown in Figures12 and 14. Finally, the right side set bolt broken out by tension failure mode and the upper plate drop off from the bearing parts.

#### 3.1.2 Perpendicular bridge axis horizontal loading (Case 2)

**Figure 15:**
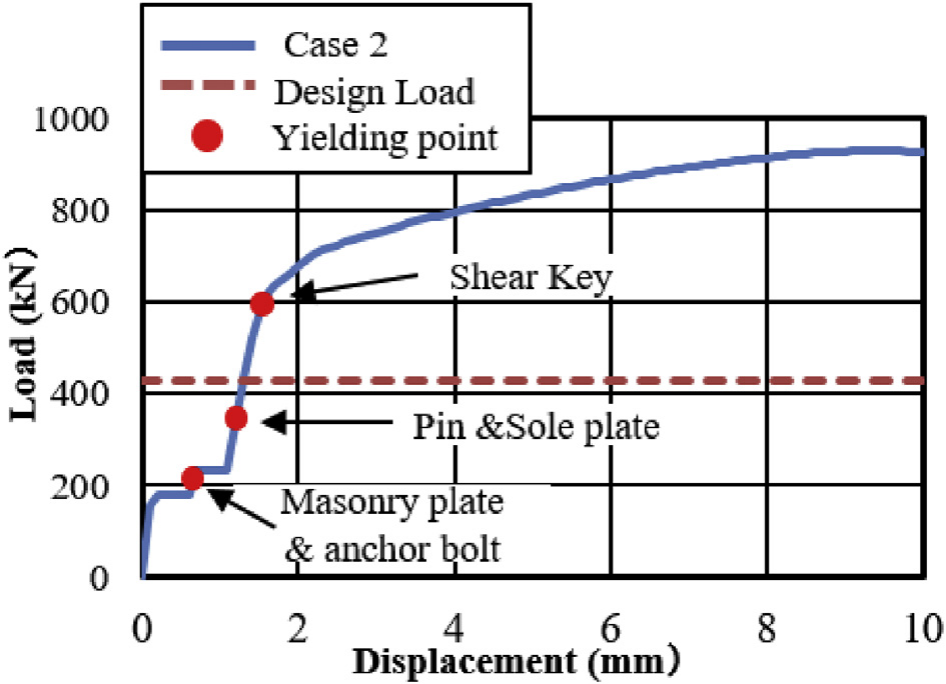
Load-Displacement curve (case 2).

**Figure 16:**
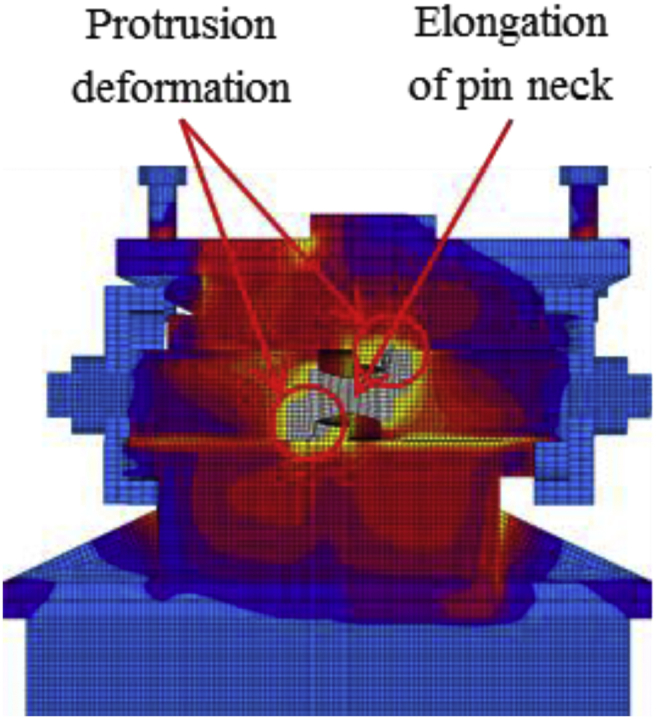
Deformation performance.

**Figure 17:**
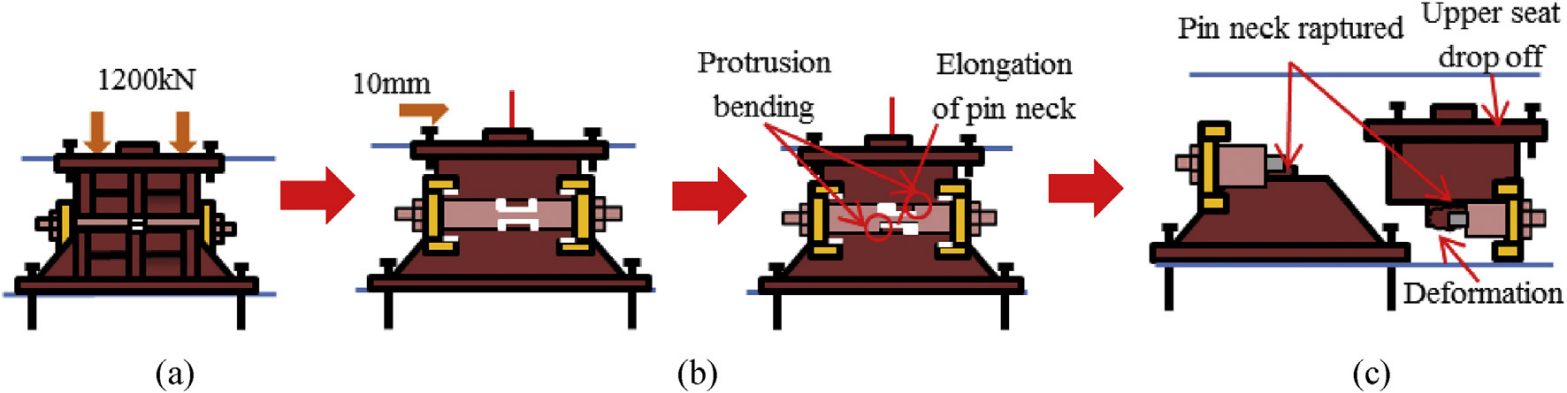
Failure process (Case 2): (a) Initial loading status; (b) Constant dead load and horizontal loading; (c) Failure final status.

The load-displacement relationship introduced by horizontal perpendicular bridge axis loading as shown in Figure 15. From the figure, the maximum load capacity of the bearing is 920kN, and yielding introduced below the expected design load except the shear key. In this direction, the horizontal load resistance capacity of the bearing depends on the set bolt and shear key of the sole plate. Contact of steel pin with upper and lower weir confirmed at 230KN, instantly after the contact lower plate and anchor bolt initiated plasticization. The upper plate and steel pin initiated plasticization at 330kN, and the shear key introduced yielding at 600kN. As the horizontal load increased in direction perpendicular to bridge axis, the pin neck introduced yielding and elongation at 550kN and 700kN, respectively and protrusion part of the plates exhibited bending deformation as shown in Figure 16. When the upper seat slipping increased in the direction of loading, plasticization and elongation of the pin neck increased simultaneously. Based on the deformation performance of the bearing, tension failure has occurred at the pin neck and split into two parts at the central axis of the pin as shown in Figure 17. From the figure, the failure was caused by tensile failure of the pin neck, and bending deformation of upper and lower protrusion portion. No visible damage found for the remaining parts of the bearings.

#### 3.1.3 45^0^ horizontal loading (Case 3)

**Figure 18:**
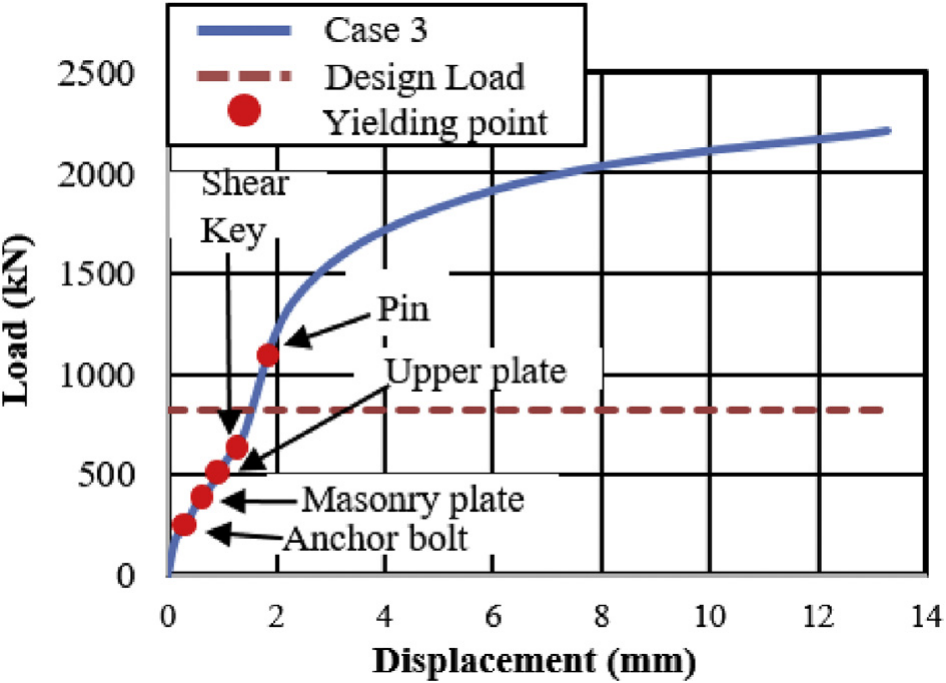
Load-Displacement curve (case 3).

**Figure 19:**
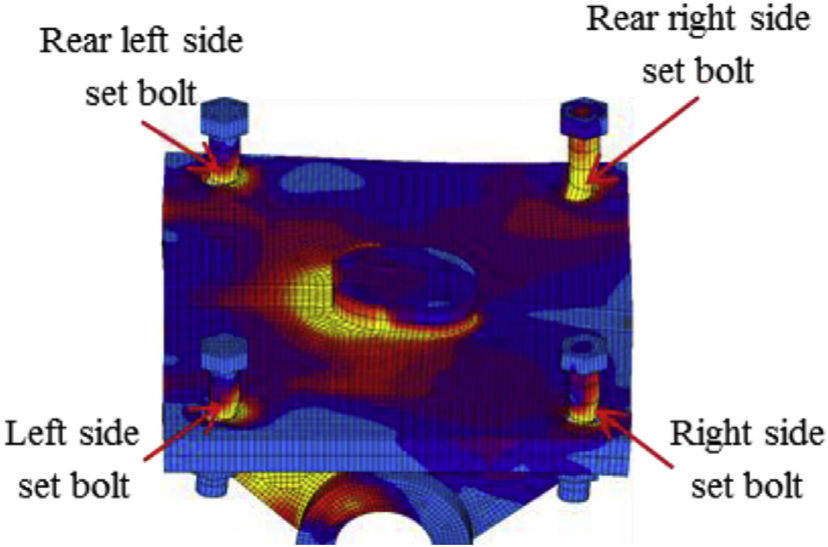
Deformation performance.

**Figure 20:**
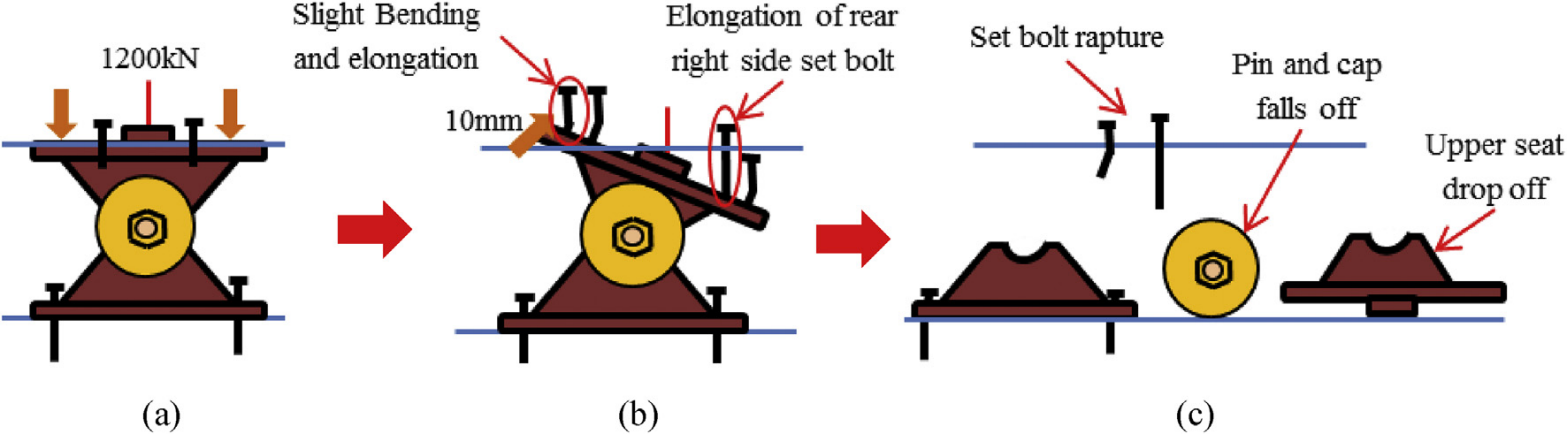
Failure process (Case 3): (a) Initial loading status; (b) Constant dead load and horizontal loading; (c) Failure final status.

Figure 18 shows the load-displacement relationship obtained from the analysis. From the relation, the maximums load capacity of the bearing is about 2200kN, and yielding generated below the expected design load except the steel pin. Contact of the solid pin with upper and lower recessed surface confirmed at 370kN, immediately after the contact the masonry plate started to slip in the bridge axis direction, and caused anchor bolt plasticization. As the horizontal loading increased in 45^0^ direction, the external force exceeded the frictional force of the upper seat and caused rotation around the pin. The upper and lower recessed surface of plates introduced yielding at 460kN and 530kN, respectively. The rotation of the upper plate increased with respect to horizontal loading, the shear key and pin introduced plasticity at 630kN and 1050kN, respectively. The deformation performance of bearing shows the combination of case 1 and 2 but addition failure was performed on set bolts as shown in Figure 19. From the figure, the rear right side set bolt generated high tensile and bending deformation compared with the remaining set bolts. Based on the deformation performance of the bearing, tension failure has occurred on the rear right side set bot, and the probable failure mode of the remaining set bolts are both tension and bending. After the rear right side set blot broken out, the horizontal loading resistance capacity directly transfer to the remaining set bolts and shear key of the sole plate. In addition, anchor bolt exhibited both slight bending deformation and tension failure due to the movement of the masonry plate in the direction of the bridge axis. Based on the previous description, the rapture of rear right side set bolt, upper seat and steel pin drop off from the bearing part are some of the failure modes of the bearing as shown in Figure 20. No visible deformation was found in the remaining parts of the bearing.

#### 3.1.4 Bridge axis horizontal and lifting load (Case 4)

**Figure 21:**
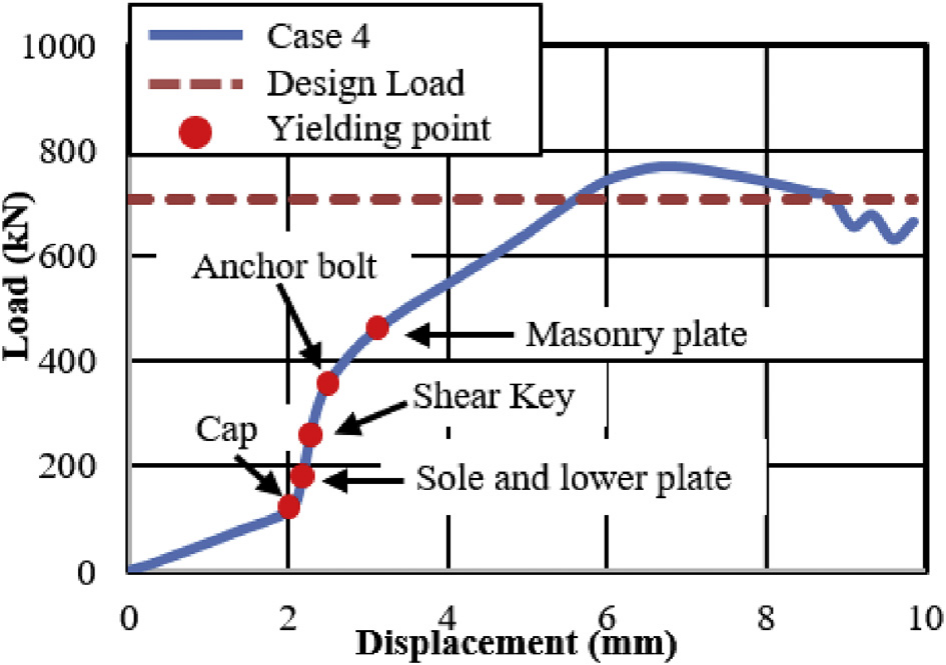
Load-Displacement curve (case 4).

**Figure 22:**
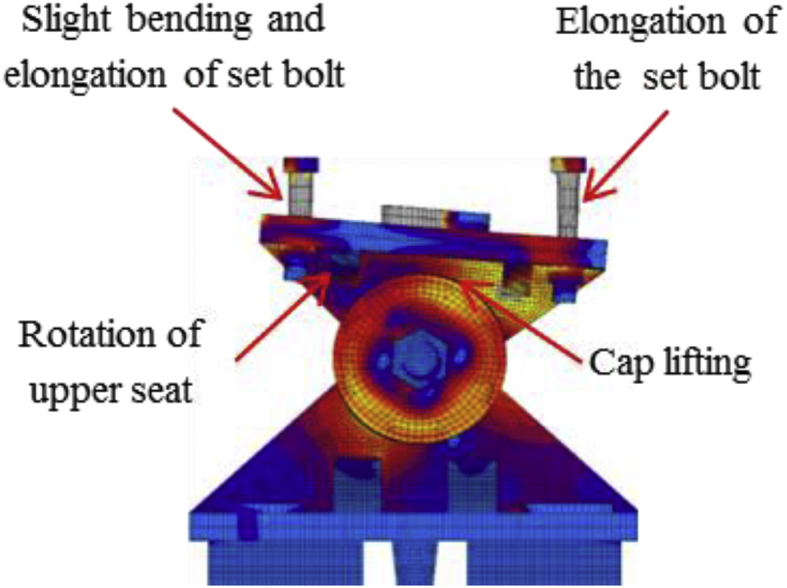
Deformation performance.

**Figure 23:**
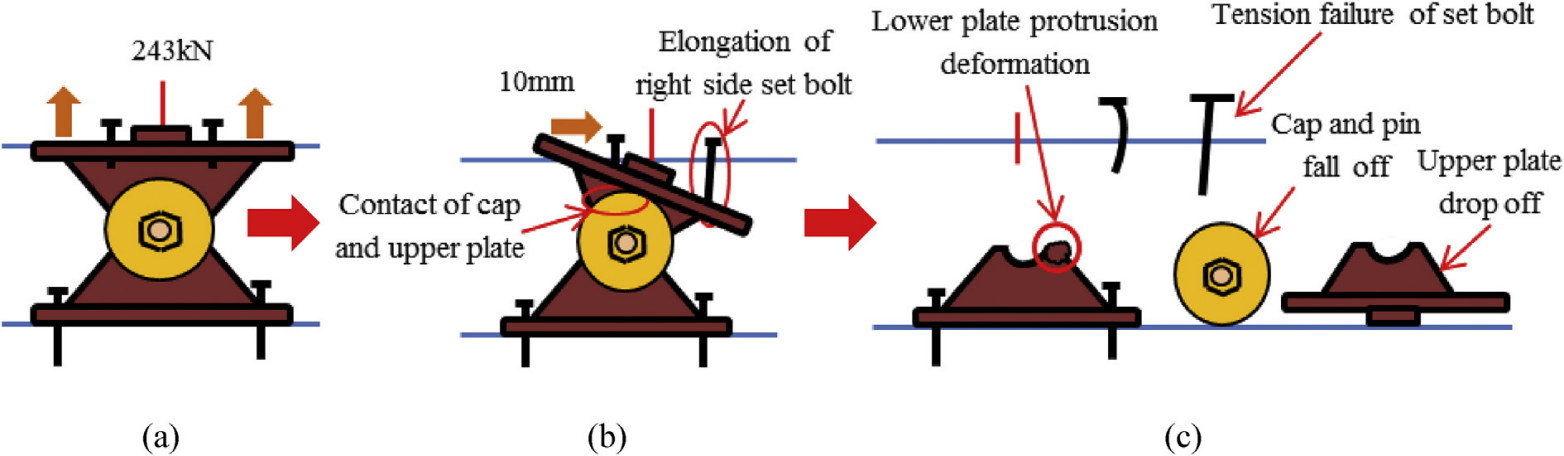
Failure process (Case 4): (a)Initial loading status; (b) Constant uplift load and horizontal loading; (c) Failure final status.

Figure 21 shows the load-displacement relationship obtained from the analysis. From the curve, it can be seen that the maximum load capacity of the bearing is about 770kN, and yielding introduced below the expected design load. Until 2mm, the stress distribution of the bearing is under elastic range due to the space between plates and cap. At 115kN the contact between the plates and cap confirmed, immediately after the contact the cap generated yielding. Initially, the upper seat rotated in perpendicular bridge axis direction due to the lifting force, and then rotated in both directions due to horizontal force and lifting force acted at the same time. The upper and lower plate plasticized at 175KN, and shear key subjected to yielding at 230kN. Anchor bolt and masonry plate initiated plasticization at 285kN and 425kN, respectively. As the horizontal load increased, the tension generated on the right and left side set bolts, and slight bending also occurred in the left side set bolt as shown in Figure 22. From the figure, the tensile deformation performance of the right and left side set bolt were completely different. Based on the deformation performance of the bearing, the right side set bolt broken out when it reaches maximum tension. After the right side set bolt broken out, the resistances capacity of the bearing directly transfer to the shear key and left side set bolts. Slight bending deformation of the upper plate rim confirmed due to rotation of the pin in perpendicular bridge axis direction. Finally, rotation of the upper seat and distortion of the upper and lower plate were increased and caused upper and lower plate wing portions bending deformation at the top and bottom contact interface. After this, the bending deformation increased and caused upper and lower plate deviation from the cap as shown in Figure 23.

#### 3.1.5 Perpendicular bridge axis horizontal and lifting load (Case 5)

**Figure 24:**
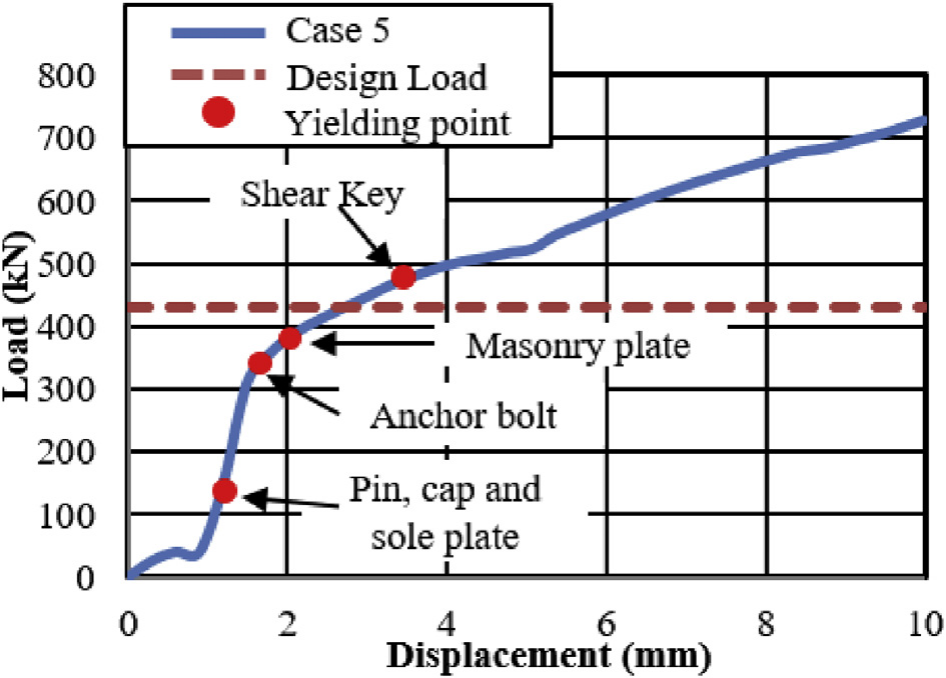
Load-Displacement curve (case 5).

**Figure 25:**
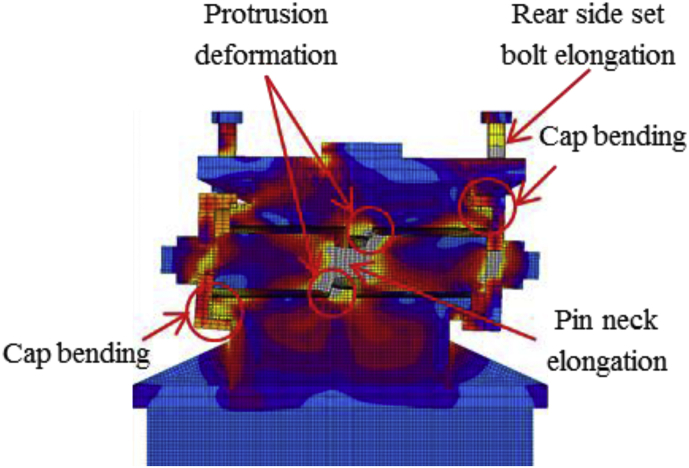
Deformation performance.

**Figure 26:**
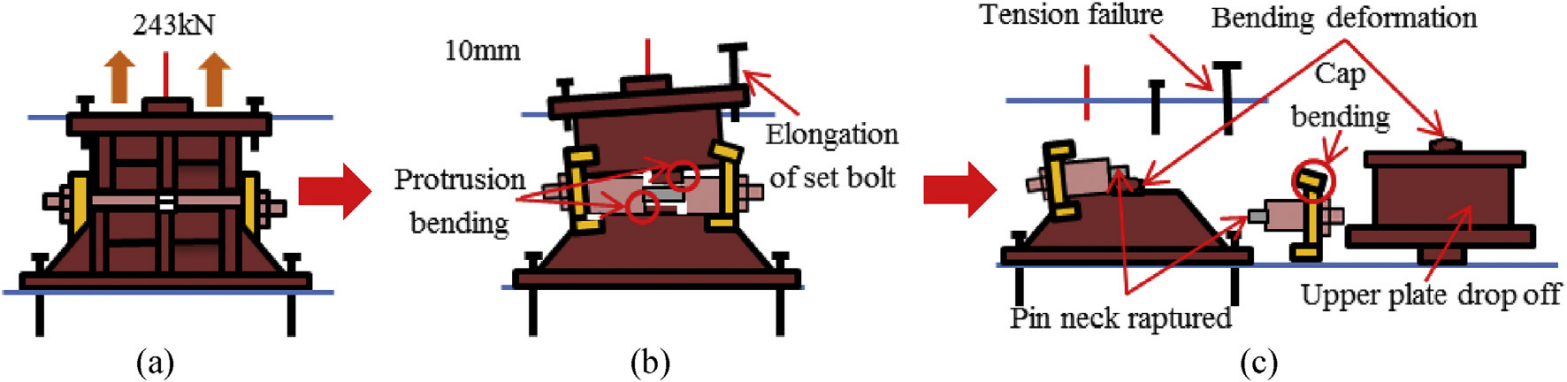
Failure process (Case 5): (a)Initial loading status; (b) Constant uplift load and horizontal loading; (c) Failure final status.

The load-displacement relationship introduced by horizontal perpendicular bridge axis loading presented in Figure 24. From the figure, it can be observed that the maximum load capacity of the bearing is 730kN and yielding introduced below the expected design load expect the shear key. At 150kN, the contact between the upper plate and cap confirmed, immediately after the contact upper and lower plate protrusion portion, steel pin, and cap initiated plasticization. As the horizontal loading increased in the direction perpendicular to the bridge axis, slipping of the upper seat was also increased and caused anchor bolt, masonry plate and shear key plasticization at 310kN, 360kN and 410kN, respectively. When slipping of the upper seat increased, the pin neck and rear (right and left) side set bolt subjected tension. Due to the horizontal and uplift force acted at the same time, the upper seat starts rotation in the bridge axis direction, and generated high contact stress on the cap (top and bottom) and upper plate wings contact interface. As a result, the upper and lower plate wings are separated by the uplift force, and the pin constriction portion and lower plate wing start to resist the horizontal force which restrained by the bottom portion of the cap. The top and bottom portion of the cap, and protrusion portion of upper and lower plates generated bending deformation as shown in Figure 25. Bending deformation of protrusion portions and elongation of the pin neck increased with respect to horizontal loading. Finally, the upper seat deviated from the cap due to the lifting force. As the horizontal displacement of the upper and lower seat increased, the pin neck and rear (right and left) side set bolt reach maximum tension. After this tension, failure has occurred at the central axis of the pin neck, and the pin separates into two parts as shown in Figure 26.

### 3.2 Pin-roller bearing

#### 3.2.1 Bridge axis horizontal loading (Case 1)

**Figure 27:**
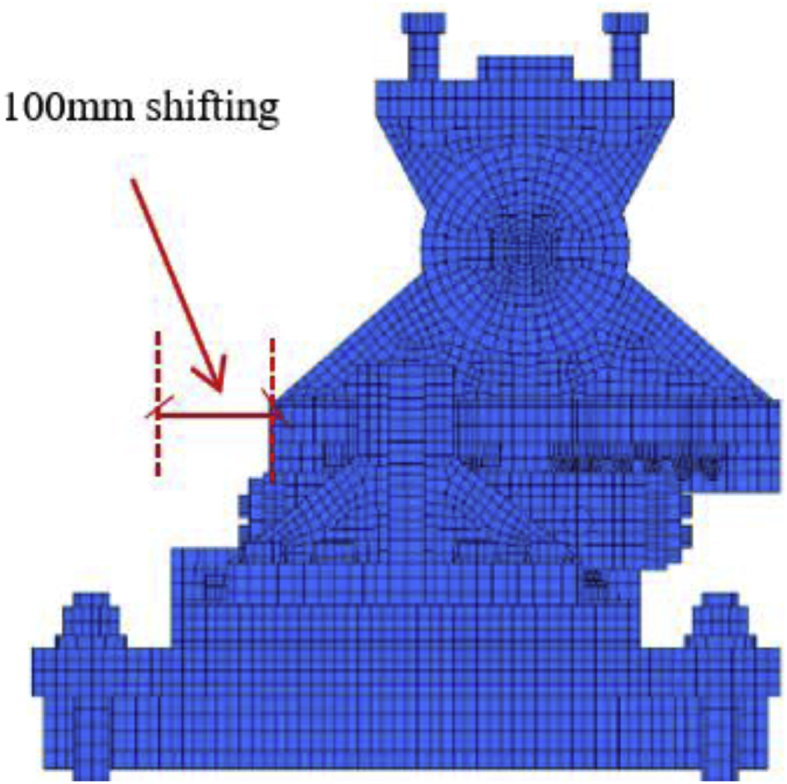
100mm shifting.

**Figure 28:**
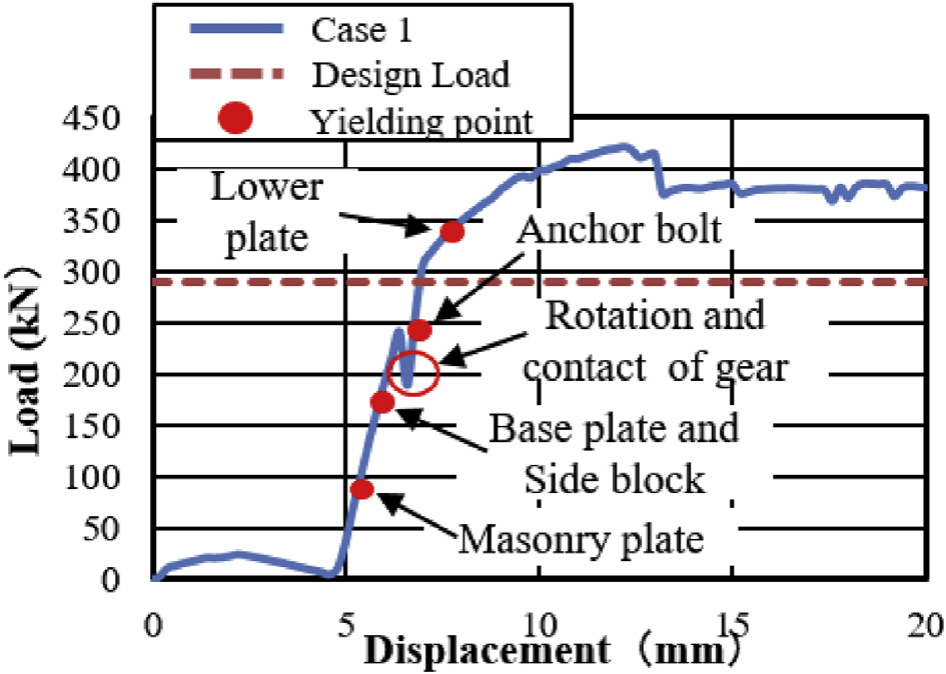
Load-Displacement curve (case 1).

**Figure 29:**
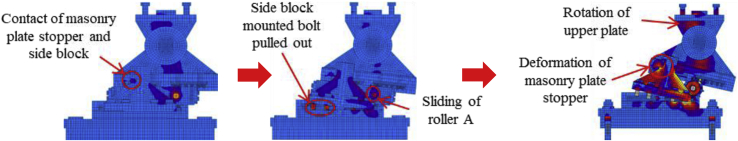
Contact and deformation performance (Case 1).

**Figure 30:**
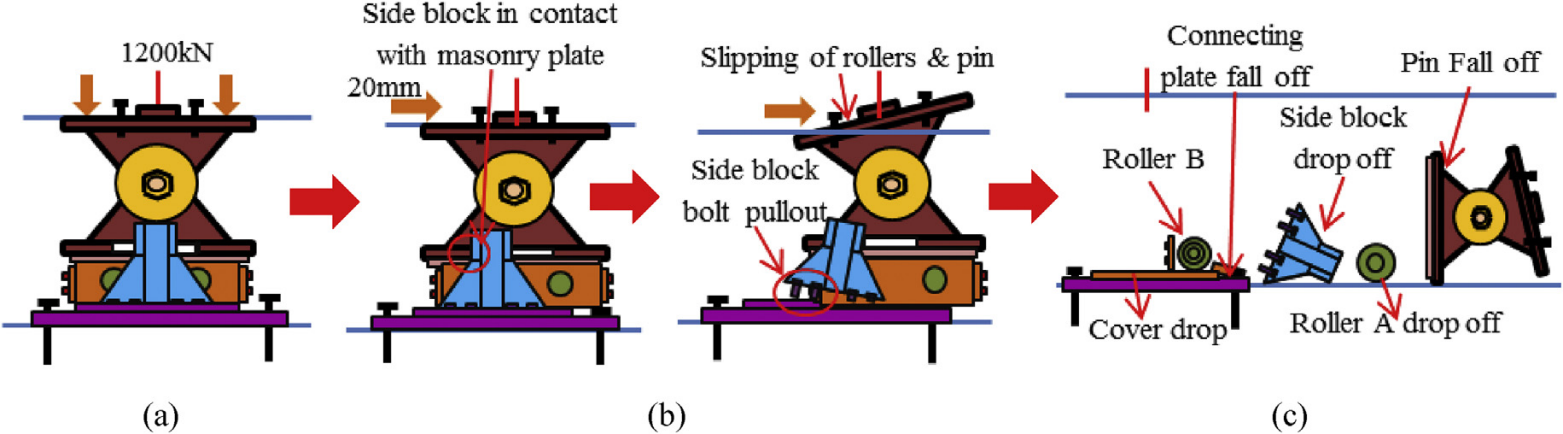
Failure process (Case 1): (a)Initial loading status; (b) Constant dead load and horizontal loading; (c) Failure final status.

In this case, the analysis carried out by consider 100mm shifting of the pin in the bridge axis direction to reduce the space between the masonry plate of the pin and side block for analysis simplicity as shown in Figure 27. The load-displacement relationship introduced by horizontal bridge axis loading displayed in Figure 28, from the figure the maximum load capacity is around 380kN and all parts of the bearing initiated yielding below expected design load except lower plate of the pin. The masonry plate stopper in contact with the side block at 70kN, immediately after the contact masonry plate, base plate and side block plasticized at 100kN and 165kN, respectively. At around 190kN the load declined due to rotation and contact of the gear. As the horizontal load increased in the direction of the bridge axis, the anchor bolt and lower plate of the pin bearing introduced plasticization at 240kN and 340kN, respectively. At the early stage of the analysis, the contact surface of steel pin and roller A initiated yielding under compression load. Due to the contact of internal parts of the bearing, the loading exhibited fluctuation and generated high pressure on the entire bearing. As the horizontal displacement of the pin and sliding of roller A increased, the side blocks started to rotate in the perpendicular bridge axis direction, and side block mounted bolt polled out from the base plate, and deformation of edge of the masonry plate stopper was confirmed as shown in the Figure 29. From the figure, the side block and side block mounted bolt subjected to bending and tension, respectively. When sliding of roller A in the loading direction, and rotation of pin and side block in the direction perpendicular to the bridge axis increased, the side block mounted bolt polled out completely from the base plate and side block failed under bending. After this, the upper seat of the pin started to the rotation against the loading direction as shown in Figure 29. The failure process of the entire bearing as shown in Figure 30, from the figure the final failure status include side block and side block mounted bolt failure, pin and roller A fall off from the bearing parts, bending deformation of masonry plate stopper, and connecting plates failure on the base plate are some of the failure mode of the bearing.

#### 3.2.2 Perpendicular bridge axis horizontal loading (Case 2)

**Figure 31:**
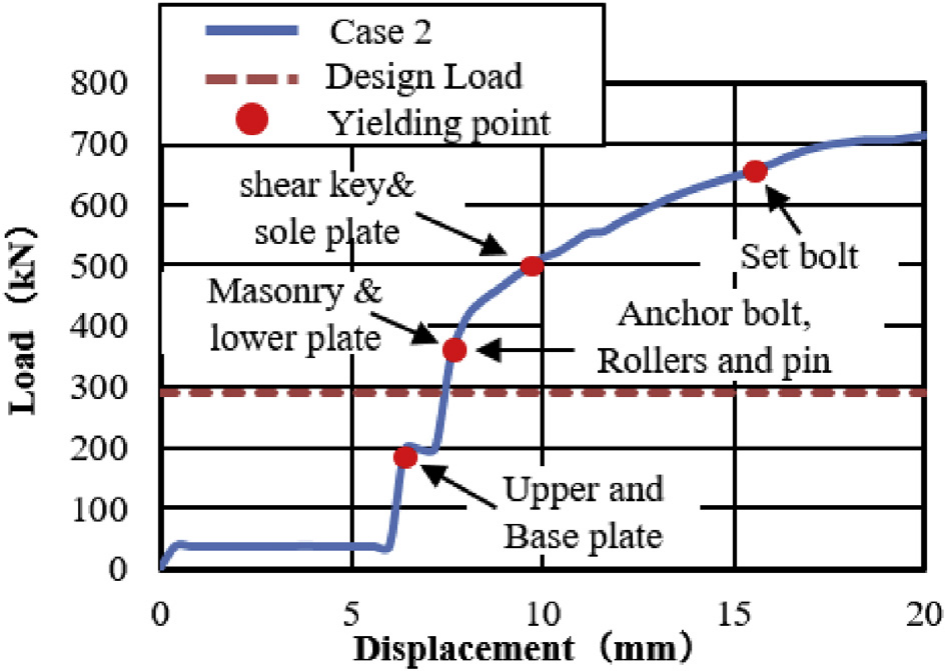
Load-Displacement curve (case 2).

**Figure 32:**
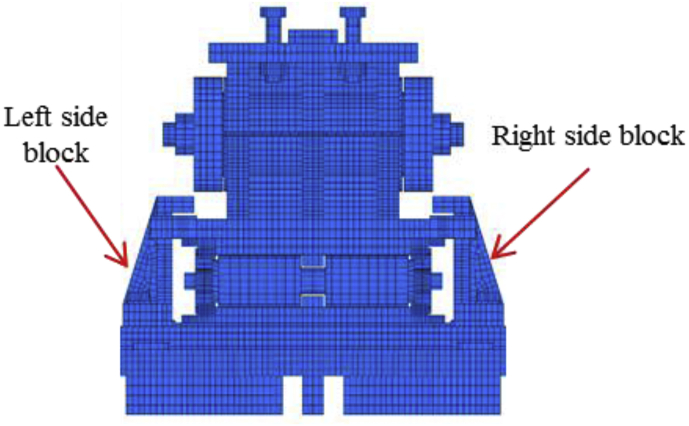
Side blocks Layout.

**Figure 33:**
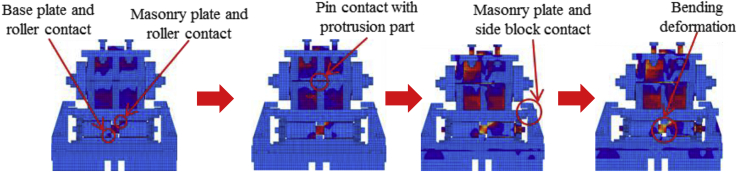
Contact and deformation performance (Case 2).

**Figure 34:**
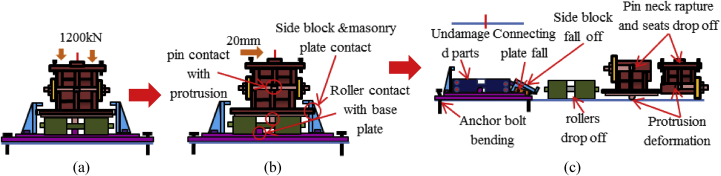
Failure process (Case 2): (a)Initial loading status; (b) Constant dead load and horizontal loading; (c) Failure final status.

The load-displacement curve using perpendicular bridge axis horizontal loading as shown in Figure 31. From the curve, it can be observed that the maximum load capacity of the bearing is about 710kN, and all parts of the bearing yield above the expected design load except the upper and base plate. At around 195kN roller contact with base and masonry plate confirmed, immediately after the contact upper and base plate initiated plasticization due to the stress generated by the contact exceeded the design strength of the plate (275MPa). As the horizontal loading increased, the steel pin in contact with upper and lower plate protrusion portion at the vicinity of 350kN, instantly anchor bolt, steel pin, roller A and B have been plasticized under high contact stress concentration. Due to increasing of pin sliding in the direction perpendicular to bridge axis, the set bolt introduced plasticization at 660kN, and also contact between masonry plate and right side block was confirmed in the vicinity of 680kN (see Figure 32). When sliding of pin and rollers farther increased, bending deformation occurred on upper, lower and base plate protrusion portion as shown in Figure 33. From the figure, the right side block sheared by the masonry plate and slightly rotated in bridge axis direction. In addition to this, the side block mounted bolt pulled out from the base plate and the steel pin start to elongate due to tension. When the pin reached maximum tension, tensile failure of pin neck, roller drop off, right side block fall off due to shear and side block mounted bolt pulled out, bending of anchor bolt and plate protrusion portion are some of the failure of the entire bearing as shown in Figure 34. No visible damage was found on the left side block and the remaining parts of the bearing.

#### 3.2.3 45^0^ horizontal loading (Case 3)

**Figure 35:**
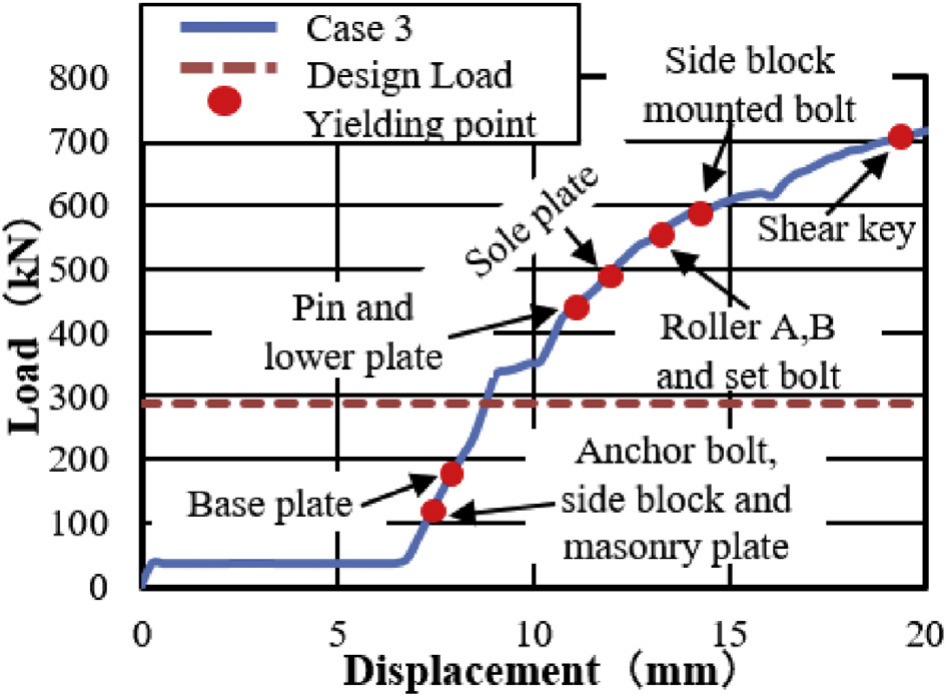
Load-Displacement curve (case 3).

**Figure 36:**
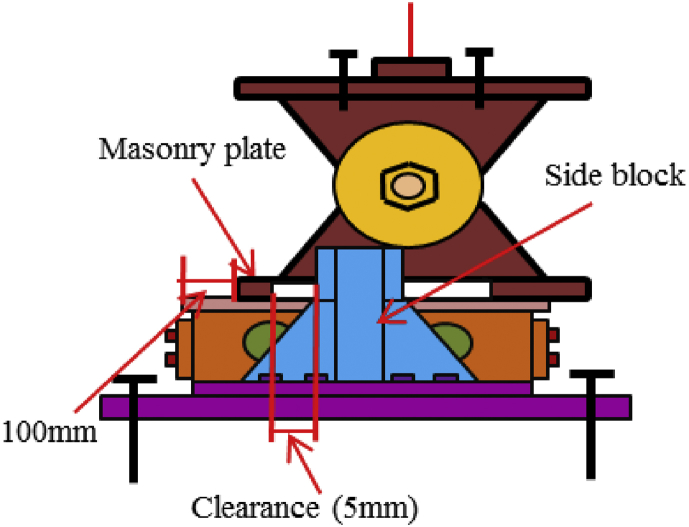
Bridge axis clearance.

**Figure 37:**
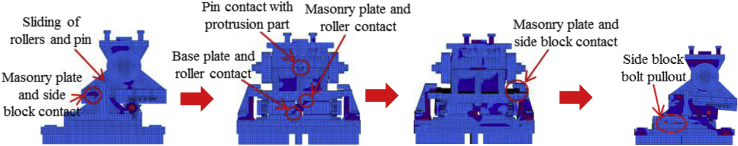
Contact and deformation performance (Case 3).

**Figure 38:**
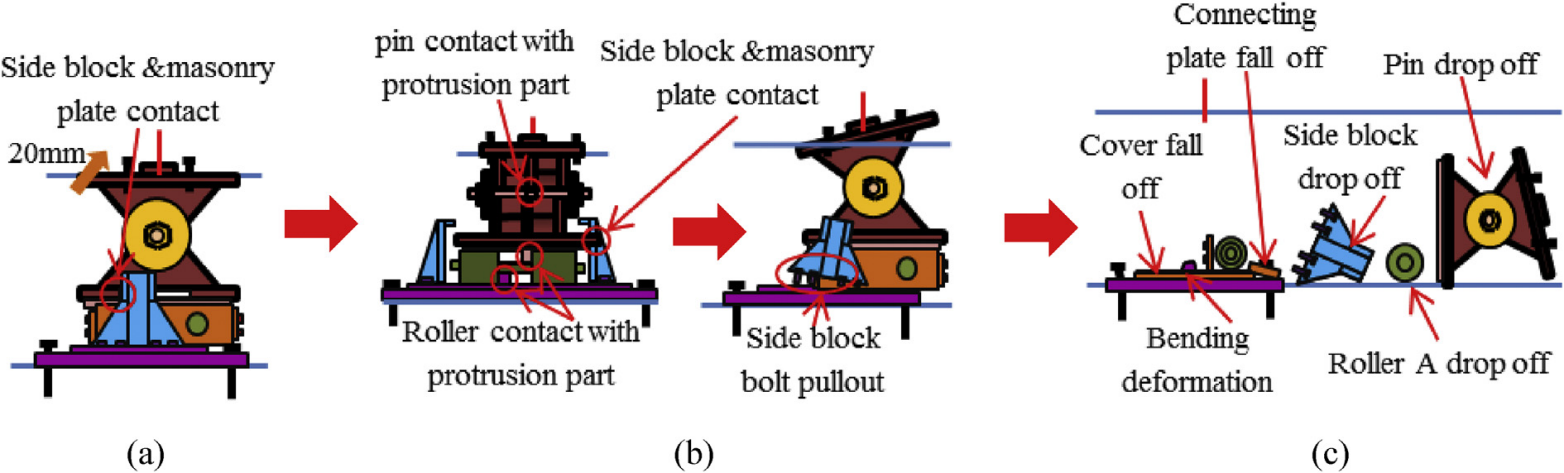
Failure process (Case 3): (a)Initial loading status; (b) Constant dead load and horizontal loading; (c) Failure final status.

In this case, the analysis performed by shifting 100mm in the direction of the bridge axis. The load-displacement curve obtained from the analysis shown in Figure 35. From the relation, the maximum load capacity of the bearing is around 750kN, and some part of the bearing plasticized below expected design load. Until 7mm, the graph shows constant load due to the space between the masonry plate and side block as shown in Figure 36. Contact of the masonry plate stopper and side block confirmed at 110kN, immediately after the contact anchor bolt and masonry plate initiated yielding at around 150kN. When horizontal load increased, the base plate and upper plate introduced plasticization at 180kN and 290kN, respectively. Contact of masonry and base plate with rollers as well as contact of steel pin with upper and lower plate protrusion portion was confirmed at 385kN and 420kN, instantly the lower plate and steel pin introduced plasticization. The stress generated at the contact interface exceeded the yield strength of the bearing parts, which caused yielding on the surface of set bolt, roller A and B, and shear key at the load of 535kN and 710kN. Figure 37 shows the contact condition and deformation performance of the bearing extracted from the analysis. From the figure, both bridge axis and perpendicular bridge axis deformation confirmed. The failure process of the bearing is similar to bridge axis failure, and some additional deformation was also confirmed (similar with perpendicular to bridge axis), which is bending deformation of base and lower plate protrusion portion, and anchor bolt as shown in Figure 38.

#### 3.2.4 Bridge axis horizontal and lifting load (Case 4)

**Figure 39:**
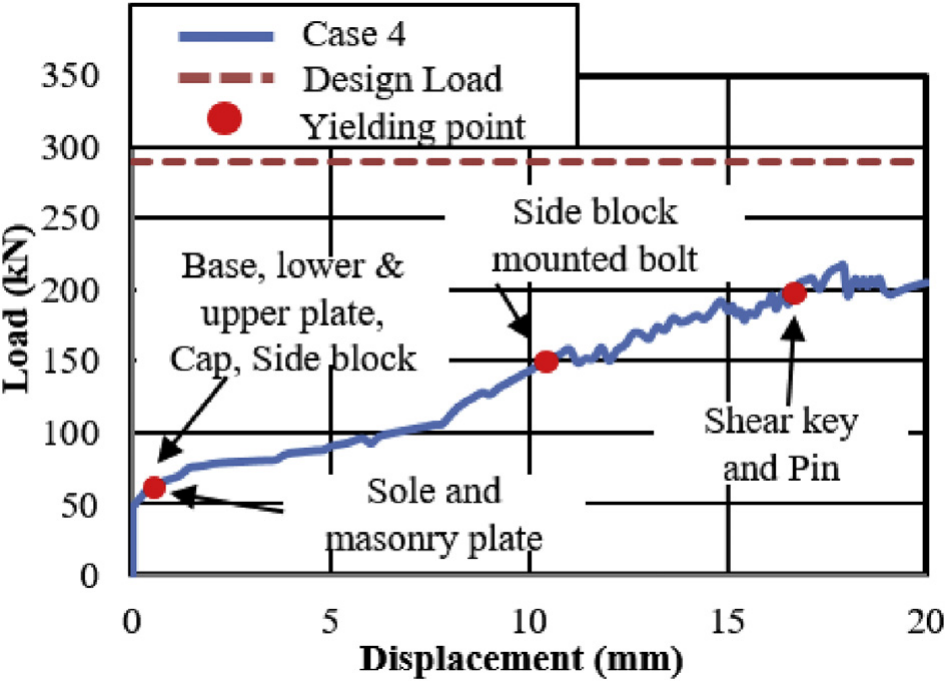
Load-Displacement curve (case 4).

**Figure 40:**
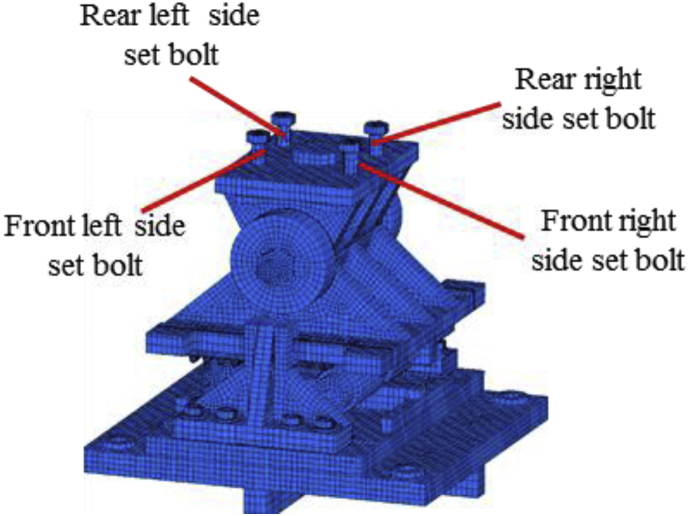
Layout of set bolts.

**Figure 41:**
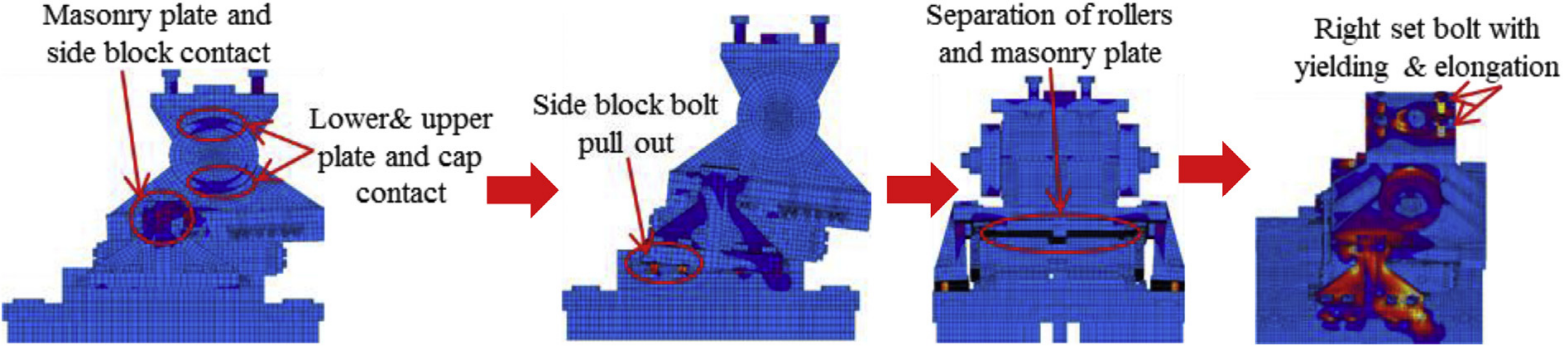
Contact and deformation performance (Case 4).

**Figure 42:**
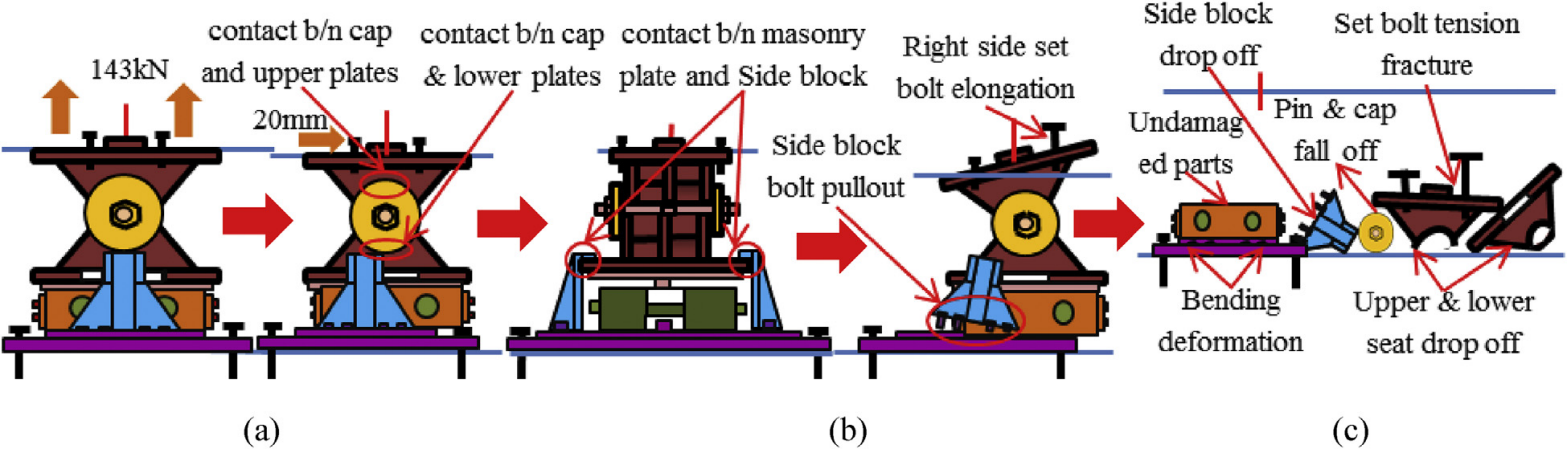
Failure process (Case 4): (a)Initial loading status; (b) Constant uplift load and horizontal loading; (c) Failure final status.

In this case, the analysis used the same model with case 1. Load–displacement relationship introduced by horizontal bridge axis loading presented in Figure 39. From the relation, the maximum load capacity of the bearing is around 210kN, and all the parts shown in the graph yield below the estimated design load. In the early stage of the analysis, contact of masonry plate stopper and side block, as well as the contact of the cap with upper and lower plate was confirmed. Under vertical loading, the contact between the masonry and base plate with rollers are completely lost, and the upper seat exhibited rotation against the horizontal loading direction (see Figure 40). As the horizontal loading increased, the side block generated high upward pressure to the main body of the bearing and caused different tensile stress concertation on the right and left side set bolts as shown in Figure 41. From the figure, the right side set bolt initiated yielding at 100kN, on the other hand, the left side set bolt introduced plasticization at 200kN. Due to the increase of horizontal loading, the side block mounted bolt and shear key initiated plasticization at 150kN and 195kN, respectively. Rotation of pin gradually increased in the direction perpendicular to the bridge axis and the side block mounted bolt start pulling out of the base plate. When the sliding and rotation of pin farther increased, the masonry plate deviated from the side block and caused side block to fall off. Upper and lower plate wings also deviated from the cap and dropout from the bearing parts. Deformation of the base plate, slight tension failure of the right side set bolt, pin and cap dropout are some of the failure modes of the bearing as shown in Figure 42, and no visible deformation found for the remaining bearing parts.

#### 3.2.5 Perpendicular bridge axis horizontal and lifting load (Case 5)

**Figure 43:**
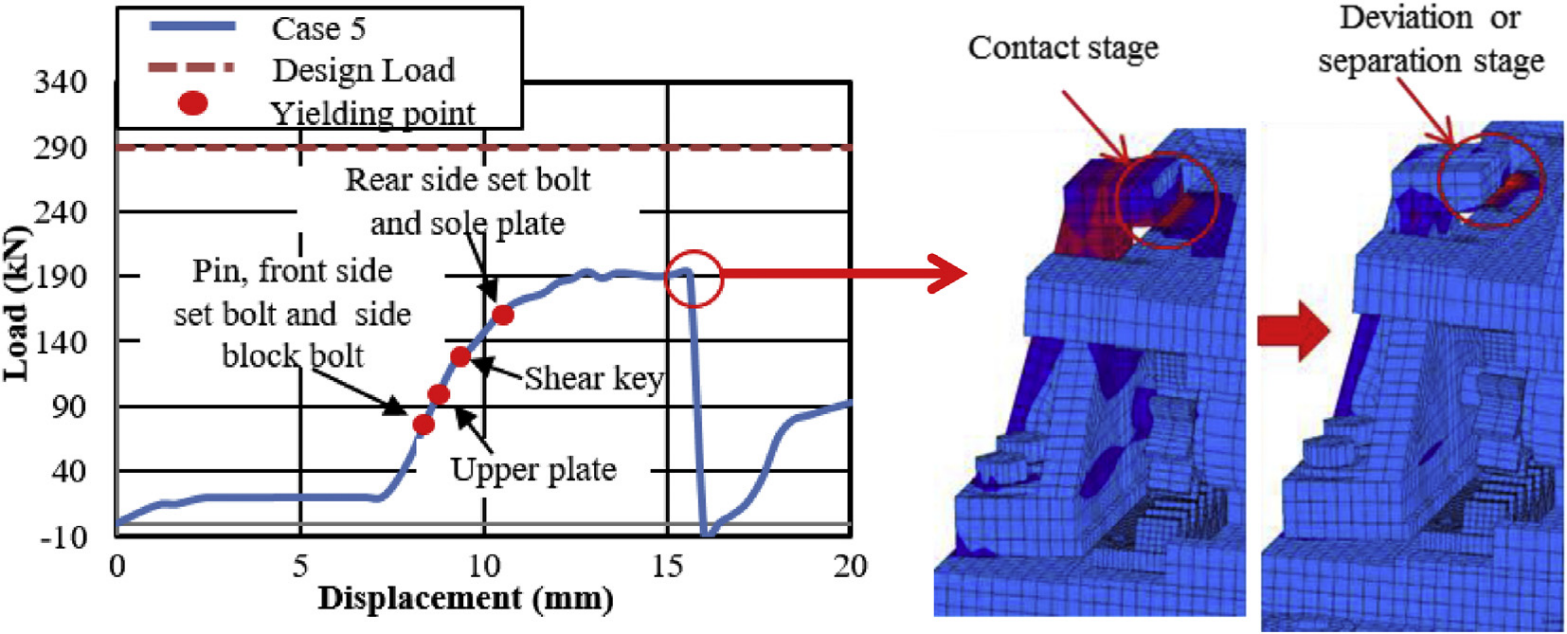
Load-Displacement curve (case 5).

**Figure 44:**
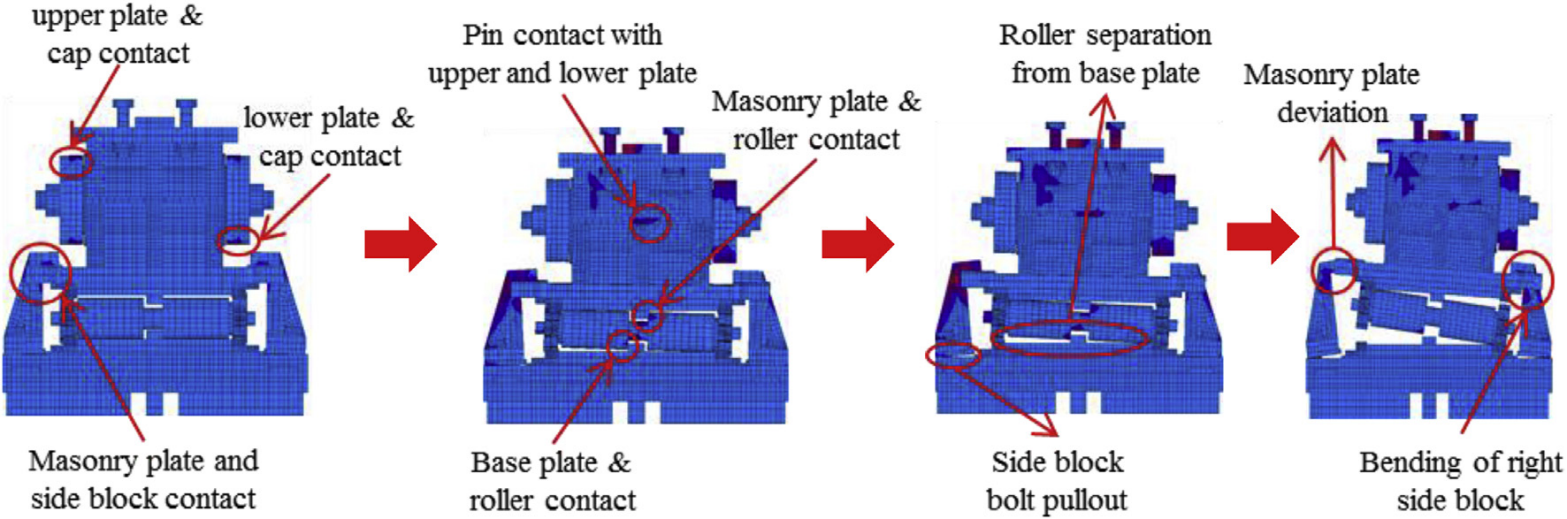
Contact and deformation performance (case 5).

**Figure 45:**
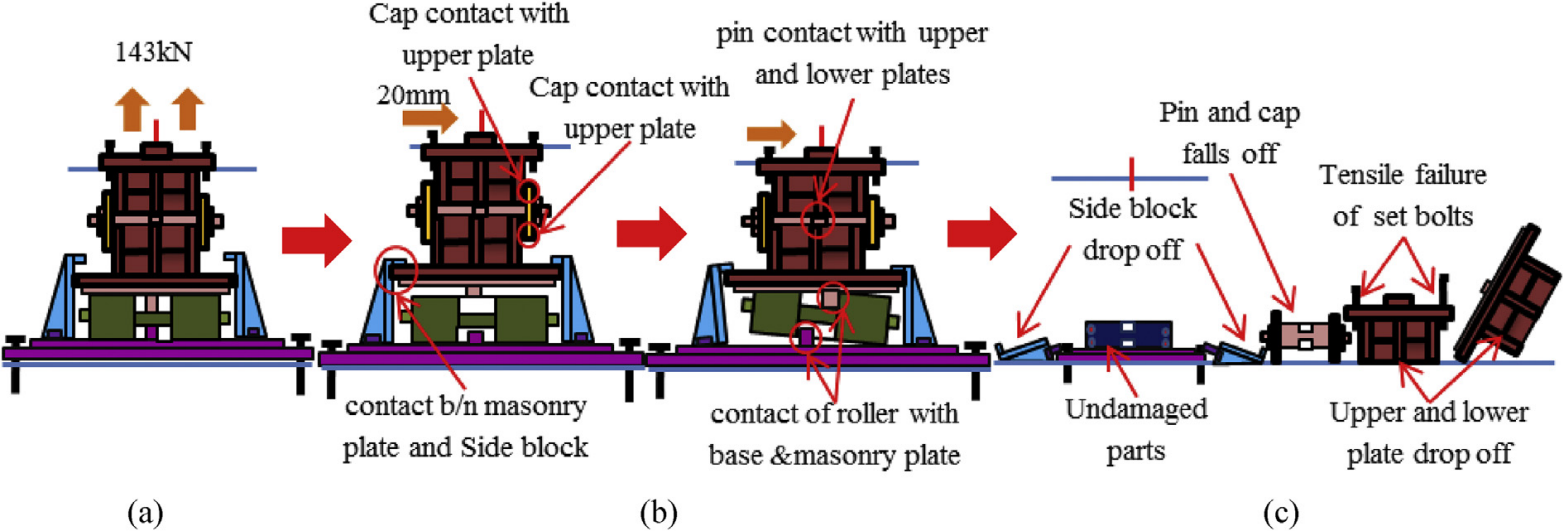
Failure process (Case 5): (a)Initial loading status; (b) Constant uplift load and horizontal loading; (c) Failure final status.

Load-displacement relationship obtained from the analysis as shown in Figure 43. From the curve, the maximum load resistance capacity is around 195kN, and all the parts shown in the graph yield below the expected design load. Under vertical loading, the majority of the bearing parts plasticized under compressive stress. When horizontal loading acted on the bearing, the pin started to slide in the perpendicular bridge direction and caused slipping of rollers. After this, uplift and horizontal loading acted simultaneously, and generate high lifting pressure and rotation on the pin at the vicinity of 50kN, instantly right side block, rear side set bolt and side block mounted bolt introduced plasticization at the load of 80kN. When the rotation of the pin increased, the bending of side block and side block mounted bolt pulled out from the based plate were confirmed at 130kN as shown in Figure 44, and caused plasticization on the base plate and shear key of the bearing. As the horizontal load increased, contact of pin with the upper and lower plate as well as the contact of rollers with base and masonry plate was confirmed at 150kN, immediately after the contact the rear (right and left) side set bolt, and sole plate generated yielding. The load declined sharply due to the deviation of the masonry plate from the left side block at 16mm as shown in Figure 43. The pin increased vertical uplift stress and bending deformation simultaneously; this caused high tensile stress concentration on the front (right and left) side set bolts and developed elongation of set bolts. The combination of tension and bending affected the side block mounted blot and caused pulling out from the base plate. Due to side block and side block mounted bolt failure, a slight deformation of the masonry plate stopper and base plate was also confirmed. Based on the deformation performance of the entire bearing, tensile failure of the set bolts, upper and lower seat drop off, pin and cap fall off and side block drop off from the bearing parts are some of the failure mode of the bearing as shown in Figure 45, and the remaining parts of the bearing are not damaged by the loading.

### 3.3 Effects of horizontal loading

#### 3.3.1 Pin bearing

**Figure 46:**
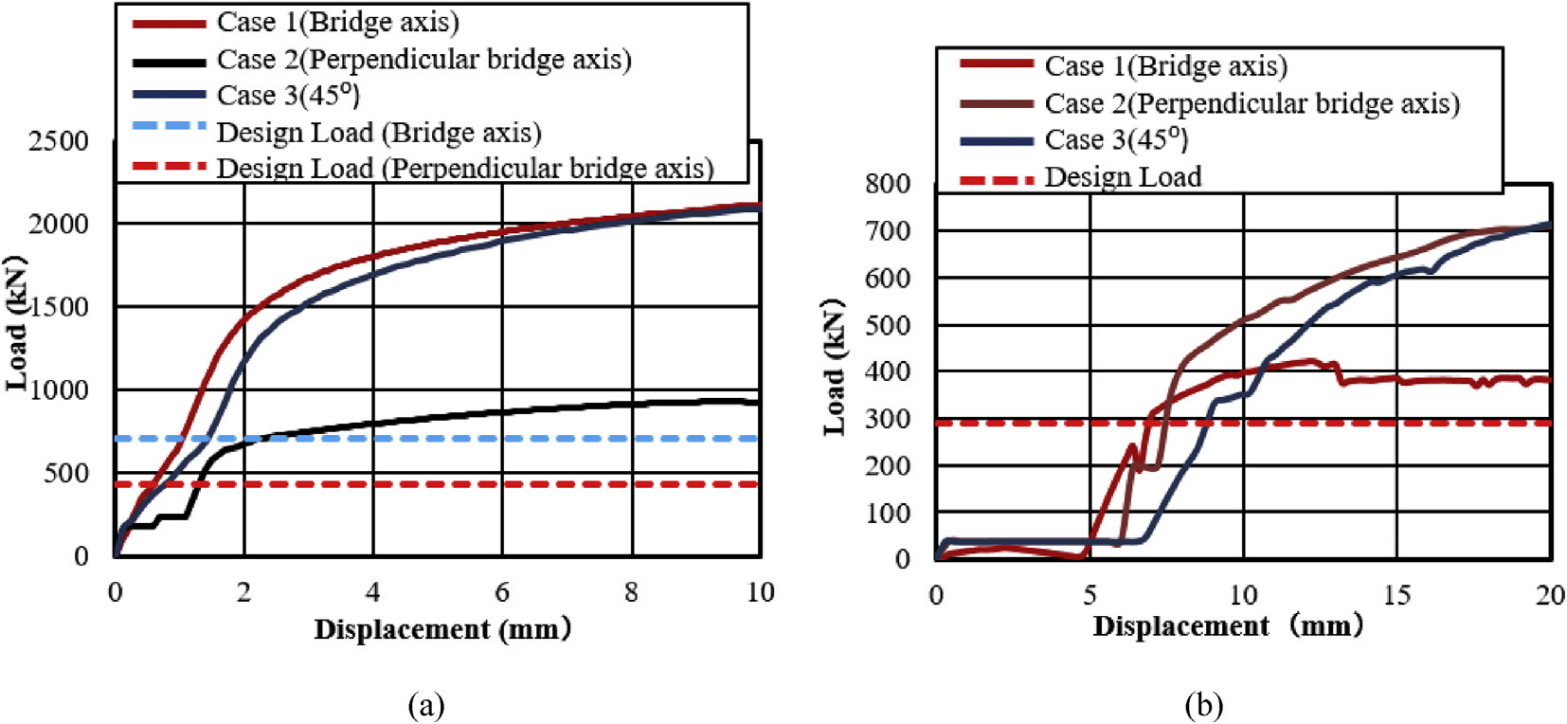
Load-displacement comparison curve: (a) Case 1, 2 and 3 of pin bearing; (b) Case 1, 2 and 3 of pin-roller bearing.

The load-displacement relationship of case 1, case 2 and case 3 as shown in Figure 46(a). From the curves, the maximum load resistance capacity of case 1 is almost the same as case 3, and the maximum load capacity of case 2 has 60 % low than the two cases due to high tensile stress concentration generated on the pin neck at early stage. The ultimate load resistance capacity of case 1 and case 3 is 68% higher than the design load estimated by Japan Road Association Design Standard; this means 2.97 times the design load of the bearing. On the other hand, case 2 is 53% higher than the estimated design load; this means 2.1 times the design load of the bearing. These show bridge axis and 45^0^ axis loading had higher resistance capacity than perpendicular bridge axis loading. From Figure 46(a), it can also observed that the load bearing capacity of the pin bearing significantly reduced when the horizontal load acting in the direction perpendicular to the bridge axis (90^0^). This results clearly show that if the horizontal load acting direction is smaller than 45^0^, it is presumed that the bearing capacity is almost the same as that of the bridge axis (0^0^). On the other hand, if the horizontal load acting direction is greater than 45^0^, it is assumed that the load bearing capacity of the bearing gradually decreases due to increment of angle.

The failure process of Case 1 and case 3 show similar deformation performance caused by rotation of the upper seat. Tension and bending failure were exhibited in the right and left side set bolt, respectively. When the right side set bolt reaches ultimate tension, tensile fracture of the set bolt occurred. Automatically, load resistance capacity of the bearing transfer to shear key and left side set bolts. There is no additional deformation was confirmed except shear key deformation found in case 1. In case 2, the pin neck greatly stretched due to slipping of the upper seat in the direction of the loading and caused high tensile stress concentration on the pin neck. When stress reaches maximum tension, the pin neck split at the center axis of the solid pin. Here, the bridge axis and perpendicular bridge axis had different failure future and mode depending on the upper seat directional deformation.

#### 3.3.2 Pin-roller bearing

The load-displacement relationship of case 1, case 2 and case 3 as shown in Figure 46(b). From the figure, the maximum load resistance capacity of case 2 is almost the same as case 3, and the maximum load capacity of case 1 has 50% low than the two cases due to high contact stress generated on masonry plate stopper and side block contact interface. The ultimate load resistance capacity of case 2 and case 3 is 61% higher than expected design load; this means 2.6 times the design load of the bearing, while case 1 is 26% higher than estimated design load; this means 1.3 times design load of the bearing. These show that perpendicular bridge axis and 45^0^ axis loading had higher resistance capacity than bridge axis loading. From the Figure 46(b), it was also found that the load bearing capacity of the pin-roller bearing significantly reduced when the horizontal load acting in the bridge axis direction (0^0^). The results apparently show that if the horizontal load acting direction is greater than 45^0^, it is assumed that the bearing resistance capacity approximately the same with that of perpendicular bridge axis (90^0^). On the other hand, if the horizontal load acting direction is smaller than 45^0^, it is inferred that the load bearing capacity of the bearing gradually decreases due to decrement of the angle.

Failure of case 1 was caused by the sliding of rollers and rotation of pin in the same way with case 3. The rotation of the pin differed for the upper and lower seat; this means the lower seat rotated in the direction of the loading, while the upper seat rotated in the opposite direction. The only additional failure for case 3 includes bending deformation of base plate protrusion part, and protective plate failure. The combination of tension and bending caused failure of bearing in case 2 beside shear. Due to the sliding of the pin and rollers in the direction of the loading, the side block sheared by the masonry plate and high tensile stress concentration generated on the pin neck. When the pin neck reached the maximum tension, the pin neck split at the central axis of the solid pin. Bending deformation of masonry and base plate protrusion were supplementary failure of the bearing in case 2. Under bridge axis and 45^0^ horizontal loading, most of the bearing parts damaged and failed compared with case 2. Failure mode of the bearing differs depend on vertical and horizontal loading direction but side block failure was the common failure mode for all cases. In general, in order to optimize the pin bearing and pin-roller bearing performance under seismic force, farther parametric analysis exploration and investigation is still necessary to understand the steel structural behavior and dynamic characteristics of the bearings.

### 3.4 Effects of vertical loading

#### 3.4.1 Pin bearing

**Figure 47:**
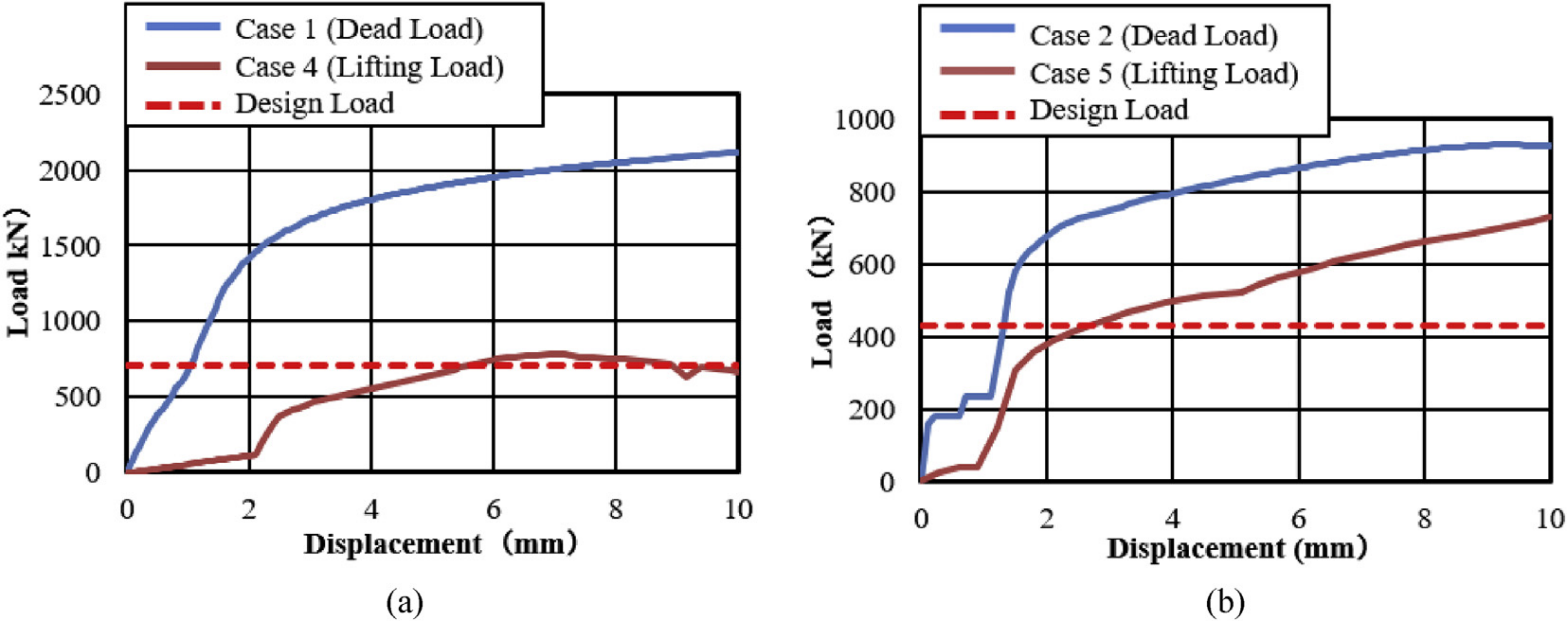
Load-displacement comparison curve of pin bearing: (a) Case 1 and 4; (b) Case 2 and 5.

Fig. 47(a) and (b) shows the load-displacement relationship of case 1 and 4 and case 2 and 5, respectively. From the figures, case 4 load resistance capacities are lower than case 1, and its closer to estimated design load of the bearing due to high contact stress generated on top and bottom of the cap at the early stage, while load resistance capacity of case 5 is relatively lower than case 2 due to upper and lower plate protrusion deformation but its higher than expected design load. The ultimate load resistance capacity of case 4 is 9% higher than the estimated design load; this means 1.1 times the design load of the bearing. On the other hand, case 5 ultimate load resistance is 41% higher than the estimated design load; this means 1.7 times the design load of the bearing. These show that the bearing had lower load resistance capacity when the bridge axis horizontal loading acted together with uplift load.

The failure process of Case 1 and 4 caused by both bending and tension failure mode. In case 1 the failure caused by rotation of upper seat, while case 4 caused by the separation of upper and lower seat from the pin due to lifting force in addition to rotation of the upper seat in perpendicular bridge axis direction. Bending deformation generated on shear key and lower seat protrusion in case 1 and case 4, respectively. For both cases, the right side set bolt and left side set bolt failure caused by tension and bending. Deformation of cap top and bottom part due to lifting force were an additional failure for case 4. Failure of case 2 and case 5 are similar except deformation of cap top and bottom part due to the upper seat lifted up by lifting force in vertical loading direction together with the cap. The bearing highly damaged when uplift load acted at the same time with horizontal bridge axis direction compared with the perpendicular bridge axis direction.

#### 3.4.2 Pin-roller bearing

**Figure 48:**
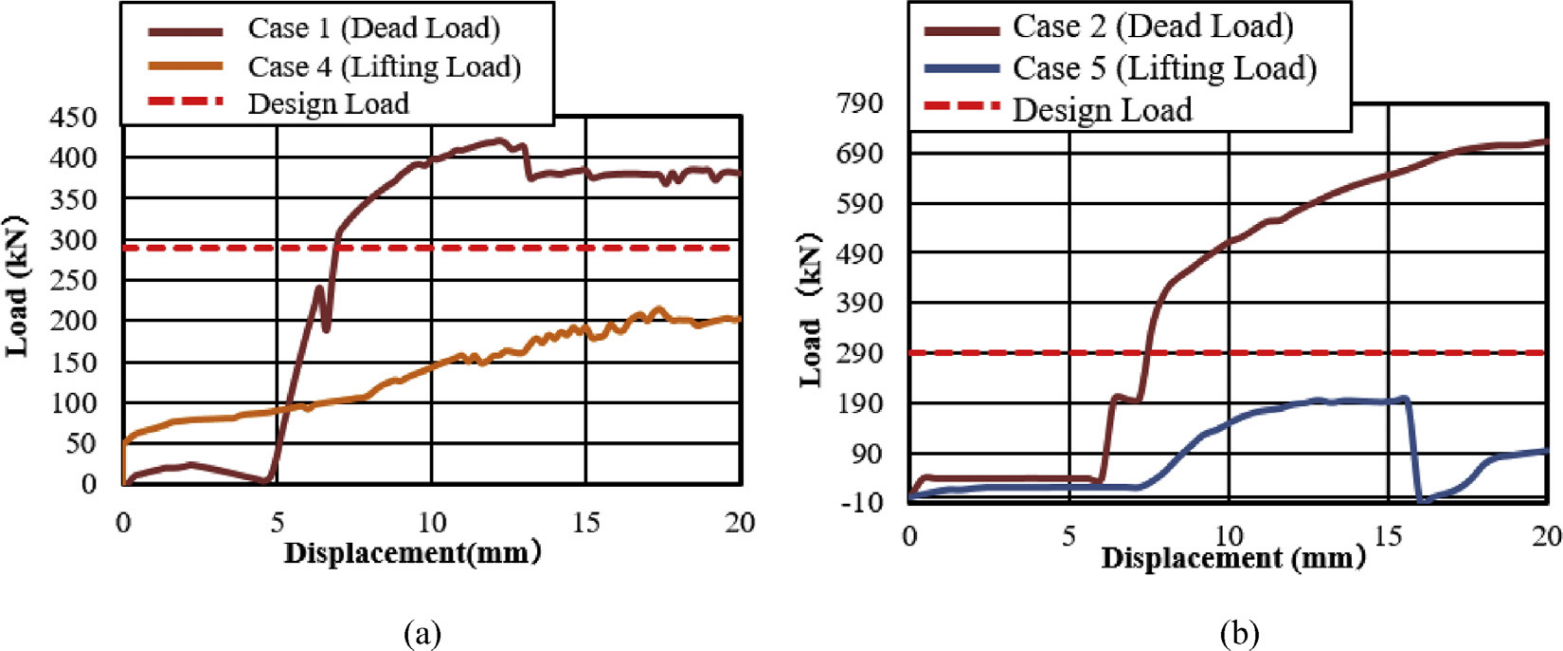
Load-displacement comparison curve of pin-roller bearing: (a) Case 1 and 4; (b) Case 2 and 5.

Fig. 48(a) and (b) shows the load-displacement relationship of case 1 and 4, and case 2 and 5, respectively. From the relation, the load resistance capacity of the bearing under lifting load is below the expected design load due to high contact stress generated between the masonry plate stopper and side blocks. Ultimate load resistance capacity of case 4 is 27% lower than estimated design load due to the contact between masonry plate and rollers was completely lost, while case 5 is 35% lower than expected design load of the bearing due to the contact between the base plate and roller was completely lost. These show that the bearing had very low load resistance capacity when both bridge axis and perpendicular bridge axis horizontal loading acted with uplift load, which means the bearing potential to resist uplift pressure is very low.

In case 1 bearing failure was initially caused by slipping of the roller and pin in the direction of horizontal loading in the same way with case 4. Bending of side block generated due to masonry plate sliding increased in case 1, while case 4 side block bending caused by the combination of slipping and lifting of masonry plate in the horizontal and vertical loading direction. The failure of each case was a different feature but most parts of the bridge damaged when the bridge axis horizontal loading acted with the dead load. Failure of case 2 as well as case 5 was caused by the combination of tension and bending. In case 2 the failure was caused by stretching of pin neck, and bending of side block, while the failure of case 5 caused by tension and bending of set bolt and side block, respectively. In case 2, roller and connected plate failure was confirmed while in case 5 steel pin and cap failure, and elongation of the set bolt was confirmed. For all cases, failure process, right and left side block failure was confirmed except case 2 only right side block was failed and no visible damage was found in the left side block. The result comparison shows that pin-roller bearing subjected to uplift loading had low horizontal load resistance capacity in bridge axis and perpendicular bridge axis direction.

## 4 Conclusion

Numerical analysis was carried out to investigate the failure process and maximum load capacity of pin bearing and pin-roller bearing based on the actual steel bearing damage caused by the 2016 Kumamoto earthquake. The analysis performed by using the 3D finite element method (FEM). The study clarifies the capacity and failure process of the bearings by considering five different combinations of loads. The main finding obtained from the analysis result as shown below.

1. For both bearing types, plasticization confirmed below the expected design load of the bearings estimated by Japan Road Association Standard. This plays a moderate role in the failure process of the entire bearings.
2. Load resistance capacity of all bearings exceeded the design load of the bearing estimated by Japan Road Association Standard except case 4 (Bridge axis with uplift load) and case 5 (Perpendicular bridge axis with uplift load) of pin-roller bearing which shows load capacity lower than the design load due to low uplift load resistance capacity of the bearing. Pin bearing also has low load residence capacity in bridge axis direction with uplift than the perpendicular axis direction.
3. In the case of pin bearing, bridge axis horizontal loading caused deformation of shear key and protrusion of lower plate, in addition, to set bolt tensile failure due to rotation of the upper seat around the pin in perpendicular bridge axis direction, and high tensile stress developed on the right side set bolt of the bearing and failed when the right side set bolt reach at maximum tension. On the other hand, perpendicular bridge axis horizontal loading caused the deformation of upper and lower plate protrusion parts besides pin neck tensile failure due to slipping of the upper seat in the loading direction. The 45^o^ horizontal loading bearing failure is the same as bridge axis failure. It was found that pin bearing had low uplift load resistance capacity, and need some design modification on protrusion of upper and lower plate, shear key, set bots and pin neck of the bearing.
4. In the case of pin-roller bearing, bridge axis horizontal loading induced different failure conditions based on vertical loading direction, which means major failure occurred when the bridge axis load acting together with dead load. Unique failure confirmed for each vertical loading case, the dead load induced deformation of protrusion of plates, roller and connecting plate failure while uplift load cause pin and cap failure, and tension failure of the set bolt. Perpendicular bridge axis horizontal loading also generated unlike failure situation, which is roller fall off and pin neck rapture induced by dead load, and pin and cap fall off and tensile failure of the set bolt was confirmed due to uplift load. Generally, pin-roller bearing shows various failure processes depends on the direction of vertical and horizontal loading.

## Declarations

### Author contribution statement

Hagere Alemayehu Gibe: Conceived and designed the analysis; Analyzed and interpreted the data; Wrote the paper.

Hiroki Tamai & Yoshimi Sonoda: Conceived and designed the analysis; Analyzed and interpreted the data; Contributed analysis tools or data.

### Funding statement

This research did not receive any specific grant from funding agencies in the public, commercial, or not-for-profit sectors.

### Competing interest statement

The authors declare no conflict of interest.

### Additional information

No additional information is available for this paper.
